# Flexibility and constraint: Evolutionary remodeling of the sporulation initiation pathway in Firmicutes

**DOI:** 10.1371/journal.pgen.1007470

**Published:** 2018-09-13

**Authors:** Philip Davidson, Rory Eutsey, Brendan Redler, N. Luisa Hiller, Michael T. Laub, Dannie Durand

**Affiliations:** 1 Department of Biological Sciences, Carnegie Mellon University, Pittsburgh, Pennsylvania, United States of America; 2 Center of Excellence in Biofilm Research, Allegheny Health Network, Pittsburgh, Pennsylvania, United States of America; 3 Department of Biology, Massachusetts Institute of Technology, Cambridge, Massachusetts, United States of America; 4 Howard Hughes Medical Institute, Massachusetts Institute of Technology, Cambridge, Massachusetts, United States of America; 5 Department of Computer Science, Carnegie Mellon University, Pittsburgh, Pennsylvania, United States of America; Indiana University, UNITED STATES

## Abstract

The evolution of signal transduction pathways is constrained by the requirements of signal fidelity, yet flexibility is necessary to allow pathway remodeling in response to environmental challenges. A detailed understanding of how flexibility and constraint shape bacterial two component signaling systems is emerging, but how new signal transduction architectures arise remains unclear. Here, we investigate pathway remodeling using the Firmicute sporulation initiation (Spo0) pathway as a model. The present-day Spo0 pathways in Bacilli and Clostridia share common ancestry, but possess different architectures. In *Clostridium acetobutylicum*, sensor kinases directly phosphorylate Spo0A, the master regulator of sporulation. In *Bacillus subtilis*, Spo0A is activated via a four-protein phosphorelay. The current view favors an ancestral direct phosphorylation architecture, with the phosphorelay emerging in the Bacillar lineage. Our results reject this hypothesis. Our analysis of 84 broadly distributed Firmicute genomes predicts phosphorelays in numerous Clostridia, contrary to the expectation that the Spo0 phosphorelay is unique to Bacilli. Our experimental verification of a functional Spo0 phosphorelay encoded by *Desulfotomaculum acetoxidans* (Class Clostridia) further supports functional phosphorelays in Clostridia, which strongly suggests that the ancestral Spo0 pathway was a phosphorelay. Cross complementation assays between Bacillar and Clostridial phosphorelays demonstrate conservation of interaction specificity since their divergence over 2.7 BYA. Further, the distribution of direct phosphorylation Spo0 pathways is patchy, suggesting multiple, independent instances of remodeling from phosphorelay to direct phosphorylation. We provide evidence that these transitions are likely the result of changes in sporulation kinase specificity or acquisition of a sensor kinase with specificity for Spo0A, which is remarkably conserved in both architectures. We conclude that flexible encoding of interaction specificity, a phenotype that is only intermittently essential, and the recruitment of kinases to recognize novel environmental signals resulted in a consistent and repeated pattern of remodeling of the Spo0 pathway.

## Author summary

Survival in a changing world requires signal transduction circuitry that can evolve to sense and respond to new environmental challenges. The Firmicute sporulation initiation (Spo0) pathway is a compelling example of a pathway with a circuit diagram that has changed over the course of evolution. In *Clostridium acetobutylicum*, a sensor kinase directly activates the master regulator of sporulation, Spo0A. In *Bacillus subtilis*, Spo0A is activated indirectly via a four-protein phosphorelay. These early observations suggested that the ancestral Spo0A was directly phosphorylated by a kinase in the earliest spore-former and that the Spo0 phosphorelay arose later in Bacilli via gain of additional proteins and interactions. Our analysis, based on a much larger set of genomes, surprisingly reveals phosphorelays, not only in Bacilli, but in many Clostridia. These findings support a model wherein sporulation was initiated by a Spo0 phosphorelay in the ancestral spore-former and the direct phosphorylation Spo0 pathways, which are observed in distinct sets of Clostridial taxa, are the result of convergent, reductive evolution. Further, our evidence suggests that these remodeling events were mediated by changes in kinase specificity, implicating flexible pathway remodeling, potentially combined with the recruitment of kinases, in Spo0 pathway evolution.

## Introduction

**Fig 1:**
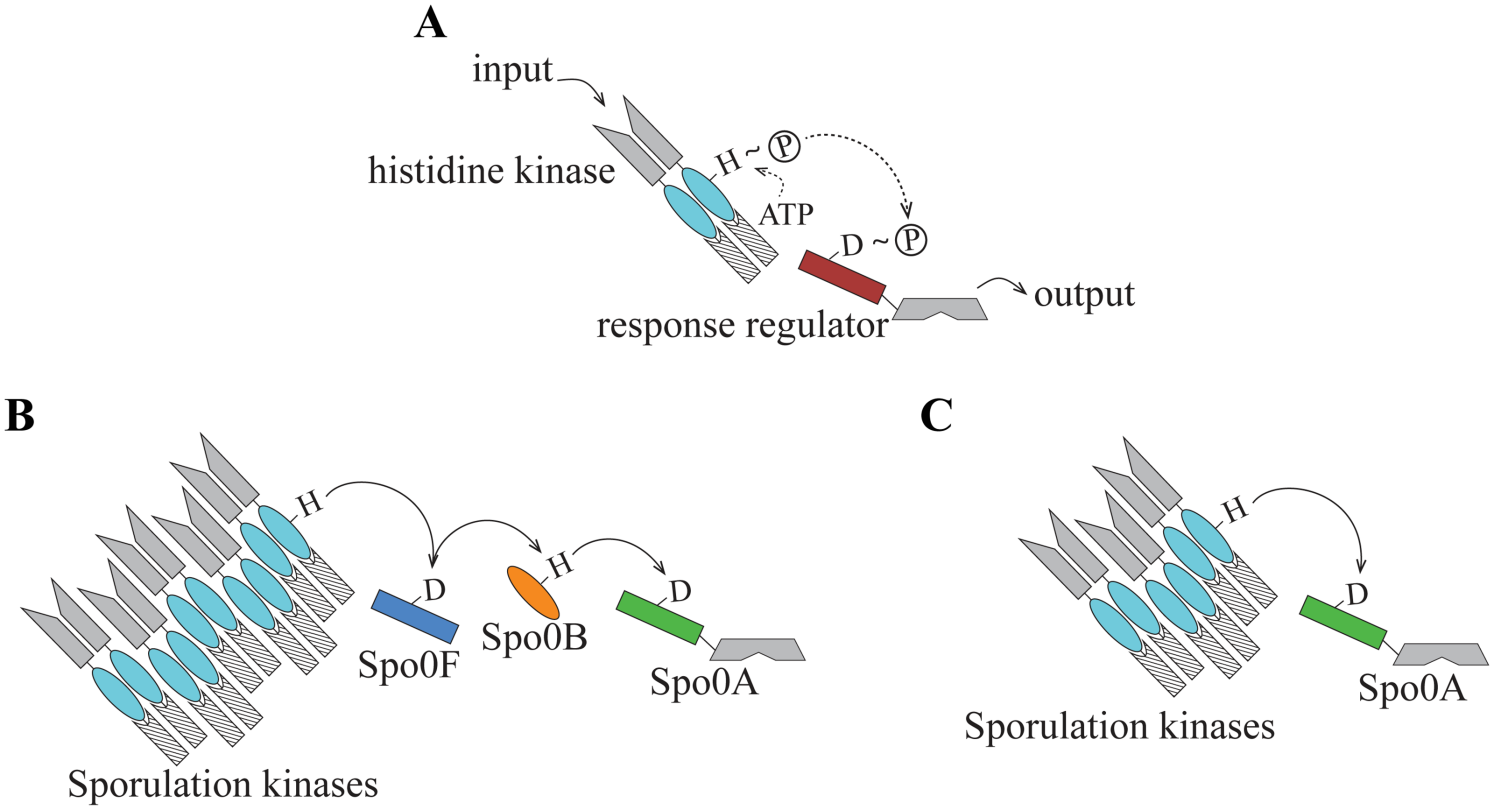
Histidine-aspartate phosphotransfer architectures. **(A)** A canonical two-component signaling system consists of a histidine kinase and a response regulator, wherein a signal is transmitted by transfer of a phosphoryl group from a conserved histidine in the HisKA domain (teal oval) to a conserved aspartate in the REC domain in the response regulator (red rectangle). **(B)** The *B*. *subtilis* Spo0 pathway is a phosphorelay. Signal transduction is initiated by activation of one of the five sensor histidine kinases that are associated with this pathway. The phosphoryl group is transferred from the HisKA domain in the kinase to the Spo0F REC domain (blue rectangle), then to the phosphotransferase Spo0B (orange oval), and finally to the REC domain of Spo0A (green rectangle). Spo0F lacks an output domain; Spo0A has the domain architecture of a typical response regulator, including a REC domain and a DNA-binding domain. **(C)** The *C*. *acetobutylicum* Spo0 pathway has a direct phosphorylation architecture, wherein multiple sporulation kinases are capable of direct transfer of a phosphoryl group to Spo0A.

Responses to changing environmental conditions are mediated by signal transduction pathways that recognize a signal, convey that signal into the cell, and initiate an appropriate cellular response. In bacteria, two-component signaling systems, typically comprised of a histidine kinase (HK) and a cognate response regulator (RR), are a primary mechanism of environmental response (Fig 1A). Signal recognition by the N-terminal sensor region of the HK leads to the autophosphorylation of a conserved histidine residue in the so-called HisKA domain by the catalytic (HK_CA) domain. The signal is then transduced by phosphotransfer from the autophosphorylated HK to a conserved aspartate residue in the N-terminal receiver (REC) domain of the RR [1]. Phosphorylation of the REC domain activates the C-terminal output domain of the RR, initiating a response to the recognized signal. Bacteria typically encode 20 to 30 two-component signaling pathways per genome [2].

**Fig 2:**
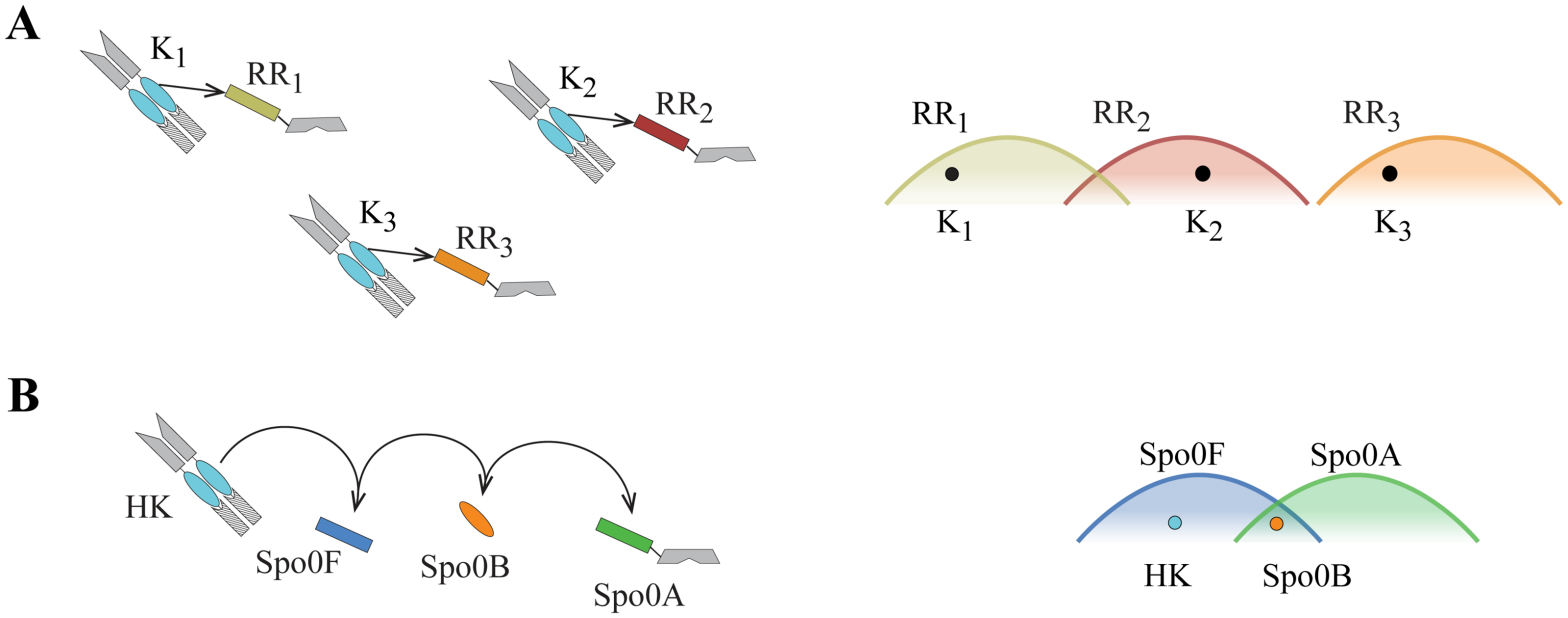
Signaling specificity and crosstalk avoidance. **(A)** Molecular recognition maintains signaling fidelity between cognate histidine kinase–response regulator pairs and prevents phosphotransfer between non-cognate proteins encoded within the same genome (e.g., two-component signaling systems shown at left). Each response regulator is capable of recognizing multiple histidine kinase specificity signatures. The set of kinase specificity signatures recognized by the response regulator is represented qualitatively as a spectrum (right). Selection likely acts to separate the specificity spectra of response regulators encoded within the same genome, resulting in little or no overlap between spectra. Each histidine kinase must occupy a non-overlapping region of the specificity spectrum of its cognate response regulator. **(B)** The requirements of signaling fidelity exert greater constraints on the specificity signatures of a phosphorelay. The phosphorelay sporulation kinase (HK) must interact with Spo0F and not Spo0A, while Spo0B must interact with both Spo0F and Spo0A (left). The phosphorelay interaction pattern requires that the spectra (right) for Spo0F (blue) and Spo0A (green) must overlap and that Spo0B (orange dot) be located in the overlapping region. Additionally, sporulation kinases (e.g., teal dot) must be located in the Spo0F specificity spectrum, but outside of the region that overlaps with the Spo0A spectrum.

A set of non-contiguous, co-evolving residues at the interface of HK and RR proteins, six in the HisKA domain and seven in the REC domain, ensure specific interaction within each cognate pair [3–6]. These specificity residues are partially degenerate: multiple sets of kinase specificity residues permit phosphotransfer to the same receiver (and vice versa [7]), such that each receiver has a spectrum of kinase specificity with which it can interact (Fig 2A). To prevent deleterious crosstalk between non-cognate proteins [8], selection acts to separate the spectra of two-component signaling pathways encoded in the same genome. Acquisition of novel pathways (*e*.*g*., through duplication or horizontal gene transfer) can cause conflicts in interaction space. The degeneracy of these interactions allows for repositioning in interaction space to eliminate crosstalk via mutational trajectories involving compensatory mutations in the cognate pair. However, in the absence of a perturbation, pathways likely remain in the same region of interaction space over the course of evolution [8].

Histidine-aspartate phosphotransfer also admits more complex signal transduction architectures. Examples include multiple-input architectures [9], multiple-output architectures [10], and so-called phosphorelays comprising a sequence of phosphotransfer interactions [11–13]. For example, the sporulation initiation (Spo0) pathway is a multi-input phosphorelay characterized extensively in *B*. *subtilis* [13–15] and also observed in closely related species [16–18]. In this architecture, multiple sensor kinases phosphorylate Spo0F, a protein possessed of a REC domain, but lacking an output domain; subsequently, that phosphoryl group is transferred via Spo0B, an intermediate histidine phosphotransferase, to Spo0A, the master regulator of sporulation (Fig 1B).

The maintenance of signal fidelity in these more complex pathways entails additional constraints on the genetic determinants of specificity because a single protein must support multiple interactions. The interaction requirements of the Spo0 phosphorelay necessitate precise molecular recognition to allow both Spo0F and Spo0A to interact with Spo0B, but only Spo0F to accept a phosphoryl group from sporulation kinases (Fig 2B). The balance of flexibility and constraint that shapes molecular recognition in these complex architectures is not well understood.

To explore this issue, we present here an analysis of the evolution of the Spo0 pathway. The Spo0 pathway controls entrance into a developmental program that produces stress-resistant, dormant endospores. The ability to produce endospores is a common feature of the Firmicutes phylum, observed in numerous species throughout two anciently related classes, the Bacilli and Clostridia, suggesting that this survival mechanism is ancient [19, 20]. These two classes are predicted to have diverged 2.7 billion years ago, coinciding with the atmospheric rise of oxygen during the great oxidation event [21]. The ancestral Firmicute was likely an obligate anaerobe, a trait that has been preserved in the present-day Class Clostridia, whereas the Bacilli are typically facultative aerobes. Many taxonomic families in both classes include both sporogenous and asporogenous species, suggesting that the ability to sporulate is frequently lost [22] through adaptation to a stable niche where sporulation is unnecessary for survival [23].

Strikingly, a comparison of the Spo0 pathways in the type species of the two Firmicutes classes, *Bacillus subtilis* [13] and *Clostridium acetobutylicum* [24], reveals that the outputs of these pathways are conserved [25], but the inputs and the signal transduction architectures are not. Spo0A, the terminal component of the pathway in both species, initiates spore development upon phosphorylation [26, 27] and is encoded by all known sporulators [22]. Spo0A is a canonical response regulator protein in its domain composition, including a REC domain [28] and a highly conserved, DNA-binding output domain, Spo0A_C [29]. Unlike Spo0A, which is likely orthologous in these distantly related species, the upstream signal transduction architectures are different. In contrast to the *B*. *subtilis* multi-input phosphorelay Spo0 architecture, *C*. *acetobutylicum* and other closely related species possess a multi-input architecture in which Spo0A is directly phosphorylated by multiple kinases [24, 30, 31] (Fig 1C).

Considering that these two different signal transduction architectures both orchestrate the initiation of sporulation through the phosphorylation of an orthologous regulator, they likely arose from a common ancestral pathway. How, then, did different signaling architectures evolve in present day species? The prevailing view is that the ancestral Spo0 pathway had a two-component direct phosphorylation architecture and the more complex phosphorelay observed in *B*. *subtilis* is a derived state [32–34]. This hypothesis was inspired by the apparent lack of Spo0F and Spo0B orthologs in the first *Clostridium* genome sequenced [32]. The simplicity of the direct phosphorylation architecture and the similarly anaerobic lifestyles of the ancestral Firmicutes and present-day Clostridia, taken together, provided further support for predictions that the original Spo0 pathway also functioned through direct phosphorylation [33]. It was further proposed that the phosphorelay likely arose in the Bacillar lineage, possibly as the result of duplication of a cognate HK-RR pair [35], and that the additional points of control associated with a phosphorelay may have contributed to adaptation to rising oxygen levels in early Bacilli [36].

Regardless of the status of the ancestral pathway, some combination of gains and losses of interaction must have occurred to produce the distinct pathway architectures observed in present day species. We took advantage of the dramatic increase in the number of sequenced Firmicutes genomes available to investigate these remodeling events. Our results challenge the prevailing hypothesis. *In silico* analyses, combined with *in vitro* experimental verification of a Clostridial phosphorelay, reveal that phosphorelay architectures are present throughout the Firmicutes. Further, we demonstrate that interaction specificity of representative Bacillar and Clostridial phosphorelays is functionally conserved. In contrast to the prevailing model, our results support a scenario in which the ancestral Spo0 pathway in the Firmicutes ancestor was a phosphorelay. The phylogenetic distribution of Spo0 architectures is patchy, consistent with several independent transitions from phosphorelay to direct phosphorylation architecture. Our results further suggest that these transitions were mediated via changes in sensor kinases, while Spo0A specificity is conserved across the Firmicutes phylum. Our findings provide a framework for reasoning about the forces that act to maintain signaling fidelity in complex signal transduction pathways with multiple interactions.

## Results

The recent increase in the number of sequenced Firmicutes genomes available offers an unprecedented opportunity to investigate Spo0 pathway evolution using a comparative approach. We assembled a set of 84 whole genome sequences that are representative of the two major sporogenous Firmicutes Classes, the Clostridia and the Bacilli (S1 Table). Genomes from Class Bacilli were selected to obtain a broad representation of the taxa in this class [37]. Within the Clostridia, most of the Clostridial clusters defined by Collins *et al*. [38] are represented by at least one species. The taxonomic nomenclature within this phylum is currently in flux [39, 40]; here, we use the taxonomic nomenclature that is currently associated with the genomes in the NCBI genome database [41, 42].

**Fig 3:**
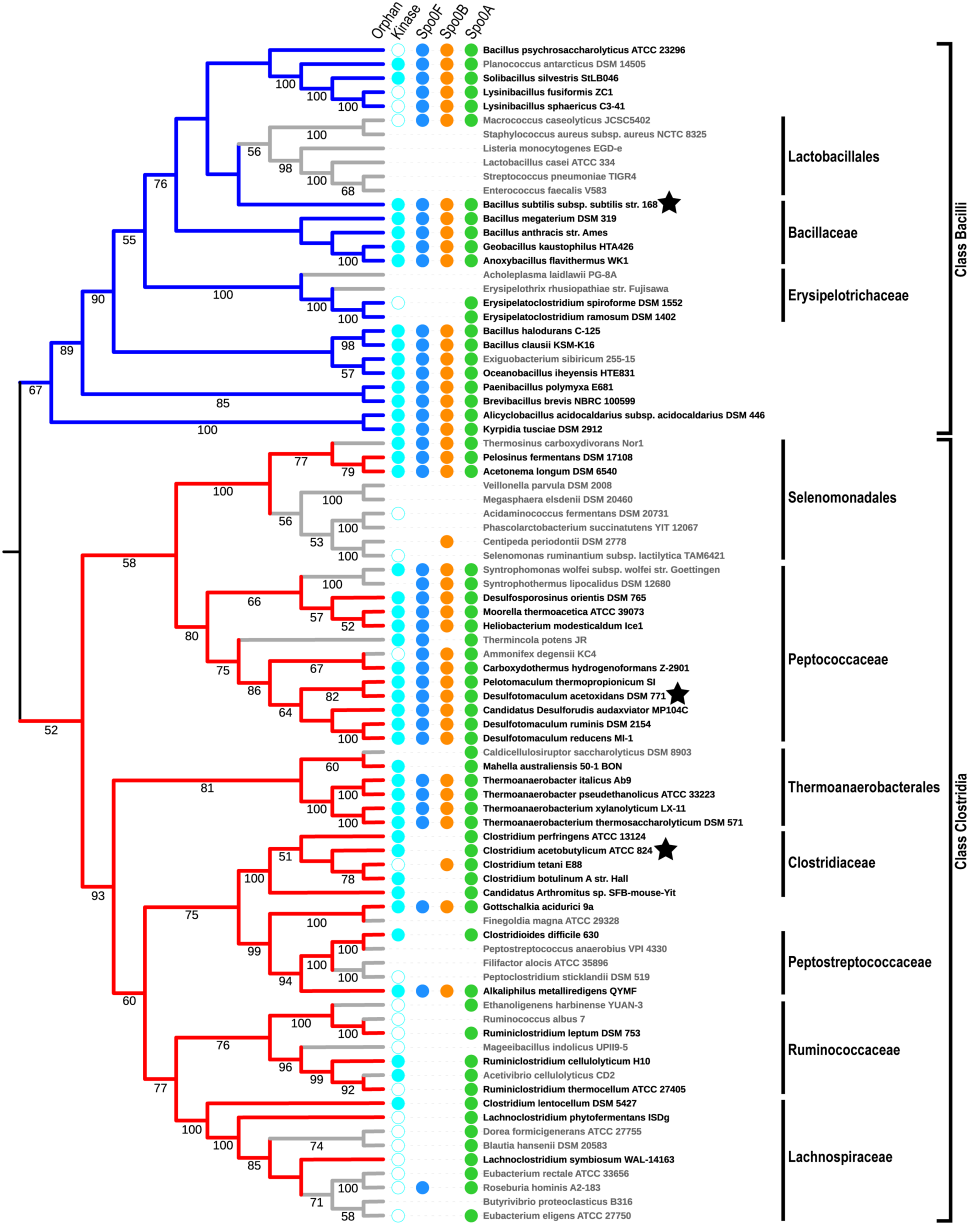
Phylogenetic distribution of predicted Spo0 pathway proteins. Cladogram of Firmicutes species used in this study, annotated with colored dots indicating predicted Spo0 pathway proteins: one or more orphan kinases (cyan); Spo0F (blue); Spo0B (orange); Spo0A (green). A filled cyan dot indicates a genome that encodes at least one orphan kinase with a PAS domain. The number of orphan kinases in each genome is given in S5 Table. Stars indicate genomes used in the *in vitro* phosphotransfer assays reported in this study. Phylogeny constructed from the concatenated alignments of 50 ribosomal protein families using RAxML with the CAT model and 100 bootstrap replicates (branch support values greater than or equal to than 50 are shown). Colored branches indicate species that are known to sporulate in Class Bacilli (blue) and Class Clostridia (red) (see also S1 Table). Species in which sporulation has not been reported are shown in grey. Tree representation created using ITOL [71]. See S1 Fig for the corresponding phylogram showing the outgroup used to root the tree and support values on all branches.

To investigate the phylogenetic distribution of genes encoding Spo0 pathway components, we constructed a maximum likelihood phylogeny for these 84 representative genomes from a concatenated alignment of 50 ribosomal proteins (Materials and methods). Ribosomal proteins have largely congruent phylogenetic signal in the Firmicutes [43], and phylogenies constructed from concatenated ribosomal protein sequences provide robust relationships in this phylum [39, 43–45]. The resulting phylogeny (Fig 3, S1 Fig) supports early divergence of Classes Bacilli and Clostridia. Further, divergence of the orders and families within each class is consistent with other phylogenies based on ribosomal proteins [43, 44]. See S1 Text and S2 and S3 Figs for a comparison of the inferred phylogenies.

### Phosphorelay architecture proteins are broadly distributed throughout sporulating Firmicutes

We undertook a survey of Spo0 components in the representative genomes to establish the architecture of modern-day Spo0 pathways. First, to establish which genomes in our representative set likely encode Spo0 pathways, we searched for response regulators encoding a Spo0A_C domain, which uniquely distinguishes Spo0A from other response regulators. We identified 68 genomes that encode an apparent Spo0A ortholog (Fig 3, green dots). Of these, 53 have been observed to form spores (Fig 3, red leaves in Class Clostridia, blue leaves in Class Bacilli; S1 Table). The presence of Spo0A in 15 apparent non-spore-formers could be due to a recent loss of sporulation or an alternate functional role for Spo0A in those species. It is also possible that these species are sporogenous, but spore formation has not been observed under the conditions tested [46].

Next, to determine the prevalence of the Spo0 phosphorelay in present-day Firmicutes, we searched for orthologs of the response regulator Spo0F and the histidine phosphotransferase Spo0B. These proteins are necessary, along with a sporulation kinase, to phosphorylate Spo0A and initiate sporulation in *B*. *subtilis* [13] and likely also in other Bacillar Spo0 pathways [16–18]. The only reported functional roles for Spo0F and Spo0B are as signal transduction intermediates in the Spo0 pathway [reviewed in 22, 47]. Thus, the presence of homologs of both Spo0F and Spo0B is strong evidence of a Spo0 pathway with a phosphorelay architecture.

Prediction of Spo0F and Spo0B homologs via sequence similarity methods has proven challenging due to the specific characteristics of Spo0F and Spo0B. Spo0F contains only a REC domain (PFAM:PF00072), making Spo0F orthologs difficult to distinguish from other response regulators that lack an output domain, such as the chemotaxis protein, CheY. Spo0B sequences lack strong sequence conservation, even within the same genus. Over broader evolutionary distances, sequence comparison cannot distinguish between Spo0B proteins and histidine kinases unambiguously [47]. PFAM domains annotated to known Spo0B homologs are either too general (SPOB_a, PFAM:PF14689) or too specific (SPOB_ab, PFAM:PF14682) to be useful identifying features for Spo0B.

Having concluded that sequence similarity and domain content do not provide sufficient information to identify phosphorelay protein orthologs, we devised an alternative method for the identification of orthologs of Spo0F and Spo0B, based on genome context. As a guide, we considered the several dozen proteins from strains of *B*. *subtilis* and its closest relatives that are annotated as Spo0F or Spo0B in the RefSeq database [48]. This guide set includes three experimentally verified Spo0F proteins [13, 17, 49] and two experimentally verified Spo0B proteins [13, 47]. Unlike many canonical two-component signaling proteins, the sporulation phosphorelay proteins are encoded in dispersed regions of the genome. The Spo0F guide set revealed that Spo0F homologs are almost always encoded immediately upstream of a fructose bisphosphate aldolase (*fbaA*) gene. Two other proteins, a CTP synthase and a transaldolase, are also commonly encoded in close proximity. Using these three genes as Spo0F neighborhood markers, putative orthologs of Spo0F were identified (S3 Table, S4 Fig). All but two spore-forming Class Bacilli genomes investigated contain a Spo0F. We also identified candidate Spo0F genes in 17 spore-forming genomes within Class Clostridia (Fig 3, dark blue dots). In contrast, 13 spore-forming Class Clostridia genomes do not encode a Spo0F-like gene in the vicinity of any of the Spo0F neighborhood markers, nor do they encode any two of the neighborhood markers in close proximity to each other. In particular, no Spo0F candidates were found in species in which a direct phosphorylation architecture has been experimentally verified (*C*. *acetobutylicum* [24], *R*. *thermocellum* [30], and *C*. *difficile* [31]). These results suggest that Spo0F homologs can be identified by conserved genome neighborhoods and are found not only in Class Bacilli, but also in many early-branching Class Clostridia taxa.

We next investigated whether genome neighborhood conservation could also be used to predict Spo0B-encoding genes. Each gene annotated as *spo0B* in RefSeq is flanked by two downstream genes encoding ribosomal proteins, L21 and L27, and an upstream gene encoding the GTPase ObgE (see also [47]). In our set of representative Firmicutes, 75 genomes encode this trio in close proximity (Fig 3, orange dots; S4 Table, S5 Fig). All but two spore-formers in Class Bacilli were found to encode a Spo0B-like protein within a five gene window that includes all three marker genes. The genomes of 18 of the 30 spore-formers within the Class Clostridia also had a region containing the three marker genes and a candidate Spo0B ortholog. The remaining Class Clostridia genomes encoded the three Spo0B neighborhood markers in close proximity, but did not encode a protein meeting the criteria of Spo0B in that vicinity. No putative Spo0B was identified in any Class Clostridia species in which direct phosphorylation of Spo0A has been verified experimentally (S4 Table). Thus, Spo0B homologs can also be identified by conservation of genome neighborhood and are found in almost all genomes in which a Spo0F homolog was identified.

In summary, we predicted Spo0F and Spo0B orthologs in most Class Bacilli genomes and in genomes broadly distributed within Class Clostridia. As the only known function of Spo0F and Spo0B proteins is phosphotransfer within the Spo0 phosphorelay, these proteins likely also perform this role in Class Clostridia species. This identification of phosphorelay proteins in multiple spore-forming taxa in Class Clostridia conflicts with the standing hypothesis that Spo0 phosphorelays, and therefore phosphorelay proteins, are restricted to Class Bacilli.

### Validation of a Peptococcaceae phosphorelay

The presence of putative Spo0F and Spo0B proteins in some spore-forming Class Clostridia species suggests that these organisms may, like those in Class Bacilli, signal the initiation of sporulation through a phosphorelay architecture. To determine whether the Spo0 proteins predicted by our method do, in fact, participate in a phosphorelay, we sought to test the *in vitro* phosphotransfer properties [50] of the putative phosphorelay proteins from *Desulfotomaculum acetoxidans DSM771* (Class Clostridia, starred in Fig 3), a spore-forming species in the Peptococcaceae [51]. The predicted homologs of Spo0F and Spo0B in this genome have conserved genomic neighborhoods. Comparison of the predicted Spo0 proteins in *D*. *acetoxidans* with their *B*. *subtilis* counterparts indicated a high degree of similarity in their respective specificity residues (Table 1, see Methods for specificity residue prediction).

**Table 1:** Predicted specificity residues in *B*. *subtilis*, *D*. *acetoxidans*, and *C*. *acetobutylicum* Spo0 proteins.

Species	Locus	Orphan Kinase Encodes PAS?	Specificity Residues			
			Orphan kinase	Spo0F	Spo0B	Spo0A
*Bacillus subtilis*	KinA	Yes	TAGFQL	QGILEVD	QLGNSL	NELLEYD
	KinB		TVGFQL			
	KinC	Yes	TSGFQI			
	KinD		TGGFQL			
	KinE	Yes	TAGFQL			
*Desulfotomaculum acetoxidans*	Dtox_0091	Yes	TTGFQM	QGILEVD	QVGLQL	NEFLDFD
	Dtox_1564		TAAFEL			
	Dtox_1918	Yes	TTGFQL			
	Dtox_2569	Yes	TTGFQM			
	Dtox_3081	Yes	TTGFQF			
	Dtox_3426	Yes	TTGFQL			
	Dtox_3834	Yes	TTGFQM			
*Clostridium acetobutylicum*	CA_C0323		NVSAQV	Not encoded	Not encoded	NEFIDYD
	CA_C0903	Yes	NISAQL			
	CA_C3319		SVGLQL			

Experimental testing of a possible *D*. *acetoxidans* phosphorelay also required prediction of the sporulation kinase(s). Experimentally verified Spo0 kinases possess few shared sequence features that definitively distinguish sporulation kinases from other sensor histidine kinases. Analysis of the regions flanking known sporulation kinases did not reveal any conservation of the genomic neighborhood. The HisKA and HK_CA domains of sporulation kinases are not markedly more similar to each other than to those of other sensor kinases, and the N-terminal sensor regions of *bona fide* sporulation kinases vary substantially. However, all experimentally verified sporulation kinases (S2 Table) are orphans, i.e., are not co-located with genes encoding other two-component signaling system proteins. Moreover, N-terminal PAS domains are observed more frequently in sporulation kinases than in the set of all kinases in the same species (S2 Table). Thus, orphan status, combined with the presence of an N-terminal PAS domain, furnishes a signature for predicting candidate Spo0 kinases. *D*. *acetoxidans* has seven orphan kinases, six of which encode a PAS domain. Strikingly, all six have putative specificity residues similar to verified *Bacillus* sporulation kinases (Table 1) suggesting that they may target Spo0F. Of these six kinases, Dtox_1918 was chosen for the phosphotransfer experiments as it has specificity residues differing from *B*. *subtilis* KinA at only one position.

To test the hypothesis that phosphotransfer to *D*. *acetoxidans* Spo0A will only be observed in the presence of a sporulation kinase, Spo0F, and Spo0B, we purified affinity-tagged variants of the four predicted *D*. *acetoxidans* Spo0 proteins (Table 2, rows 1–4; see also Methods). For the multidomain proteins (the kinase, Dtox_1918, and Spo0A, Dtox_2041), we used truncated sequences that contain the interaction domains.

**Table 2:** Protein constructs.

Protein Name	Locus id	Included residues in expression construct		Tag	Tagged Protein Size	
		Start	Stop		residues	kDa
Dt1918	Dtox_1918^1^	301	535	His_6_-MBP-TEV	652	72.0
Dt0F	Dtox_0F	1	133	TRX-His_6_-TEV	283	31.5
Dt0B	Dtox_0B	1	195	His_6_-MBP-TEV	613	68.0
Dt0A	Dtox_0A^2^	1	134	His_6_-thrombin	171	18.5
BsKinA	Bsub_KinA	1	606	His_6_-MBP-TEV	1024	115.0
Bs0F	Bsub_0F	1	124	TRX-His_6_-TEV	274	31.0
Bs0B	Bsub_0B	1	192	TRX-His_6_-TEV	342	38.5
Bs0A	Bsub_0A	1	267	TRX-His_6_-TEV	417	46.7
Ca0903	CA_C0903^3^	244	683	His_6_-MBP-TEV	857	95.0
Ca3319	CA_C3319	1	445	His_6_-MBP-TEV	863	95.5
Ca0A	CA_C0A	1	281	His_6_-thrombin	318	36.0

To increase protein solubility and stability, the constructs for Dtox_1918, Dtox_0A, and CA_C0903 encode truncated sequences that contain their respective interaction domains. Legend:

^1^: The N-terminal sensing domains were removed.

^2^: REC domain only.

^3^: N-terminal trans-membrane region was removed. His_6_: hexahistidine sequence; MBP: maltose-binding proteins; TEV: Tobacco Etch Virus nuclear-inclusion-a endopeptidase cleavage site; TRX: thioredoxin domain; thrombin: thrombin protease cleavage site. See Supplementary Text 4 for more details.

**Fig 4:**
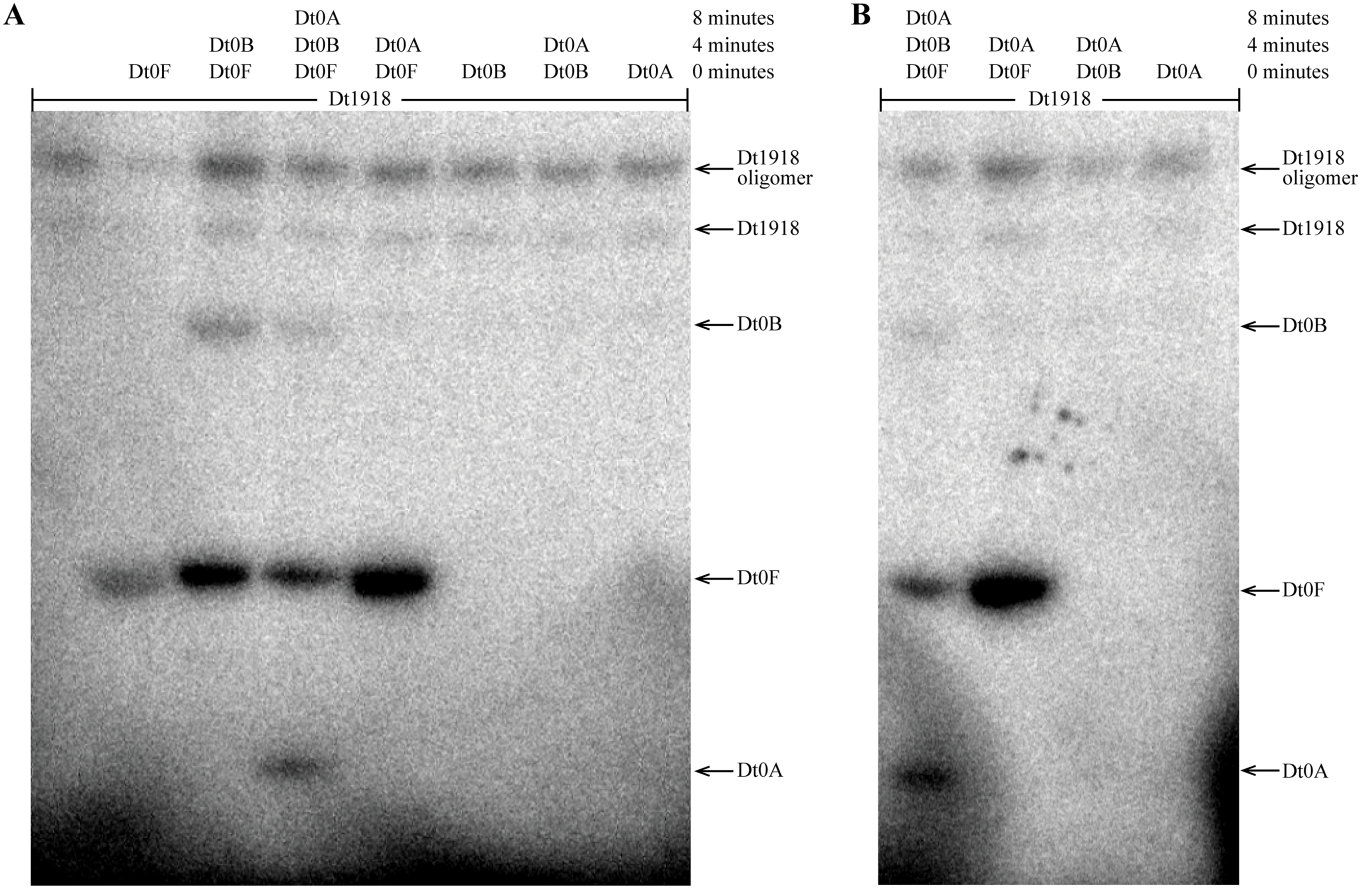
Phosphotransfer profiling of Spo0 phosphorelay proteins from *Desulfotomaculum acetoxidans*. All eight combinations of Dtox1918 and one or more downstream constituents of the predicted Dtox phosphorelay were examined for phosphotransfer (see text for details). The observed interactions are consistent with a Spo0 phosphorelay in *D*. *acetoxidans*: Spo0F and Spo0B are both necessary and sufficient for phosphorylation of Spo0A. Further, phosphotransfer to Spo0B was only observed in the presence of Spo0F. **(A)** The proteins in each reaction (listed above the corresponding lane) were added sequentially at 4 minute intervals (time points noted at right). Reactions were sampled 3 minutes after addition of the final constituent protein. **(B)** Reactions from (A) that included Spo0A were sampled again 10 minutes after addition of Spo0A. Direct phosphorylation of Spo0A was not observed even following this longer incubation period. See Table 2 for abbreviations.

To assess phosphotransfer connectivity, the purified kinase, Dtox_1918, was first incubated alone with radiolabeled ATP for 15 minutes and then examined by SDS-PAGE and autoradiography (Fig 4A, lane 1). Two bands representing autophosphorylated Dtox_1918 were seen, consistent with different kinase oligomers. Inclusion of Spo0F in the reaction (Fig 4A, lane 2) produced an additional band indicating that Spo0F can be directly phosphorylated by Dtox_1918. Similarly, the addition of Spo0F and Spo0B to autophosphorylated Dtox_1918 produced bands for each of the three proteins (Fig 4A, lane 3) and addition of Spo0F, Spo0B, and Spo0A produced bands corresponding to all four proteins (Fig 4A, lane 4). Importantly, Spo0A is only phosphorylated in the presence of both Spo0F and Spo0B (Fig 4A, lanes 5 and 7). Further, the phosphorylation of Spo0B requires the presence of Spo0F (Fig 4A, lanes 6 and 7, S8 Fig). Finally, we confirmed that Dtox_1918 cannot directly phosphorylate Spo0A under these conditions (Fig 4A, lane 8), or even following a longer incubation (Fig 4B).

Collectively, these results indicate that the Spo0F, Spo0B, and Spo0A homologs identified above, in conjunction with Dtox_1918, comprise a *bona fide* phosphorelay similar in architecture to that first characterized in *B*. *subtilis*. The *D*. *acetoxidans* phosphorelay is, to our knowledge, the first experimentally verified Spo0 phosphorelay outside of Class Bacilli.

### Evolutionarily distant Spo0 phosphorelays are functionally conserved

**Fig 5:**
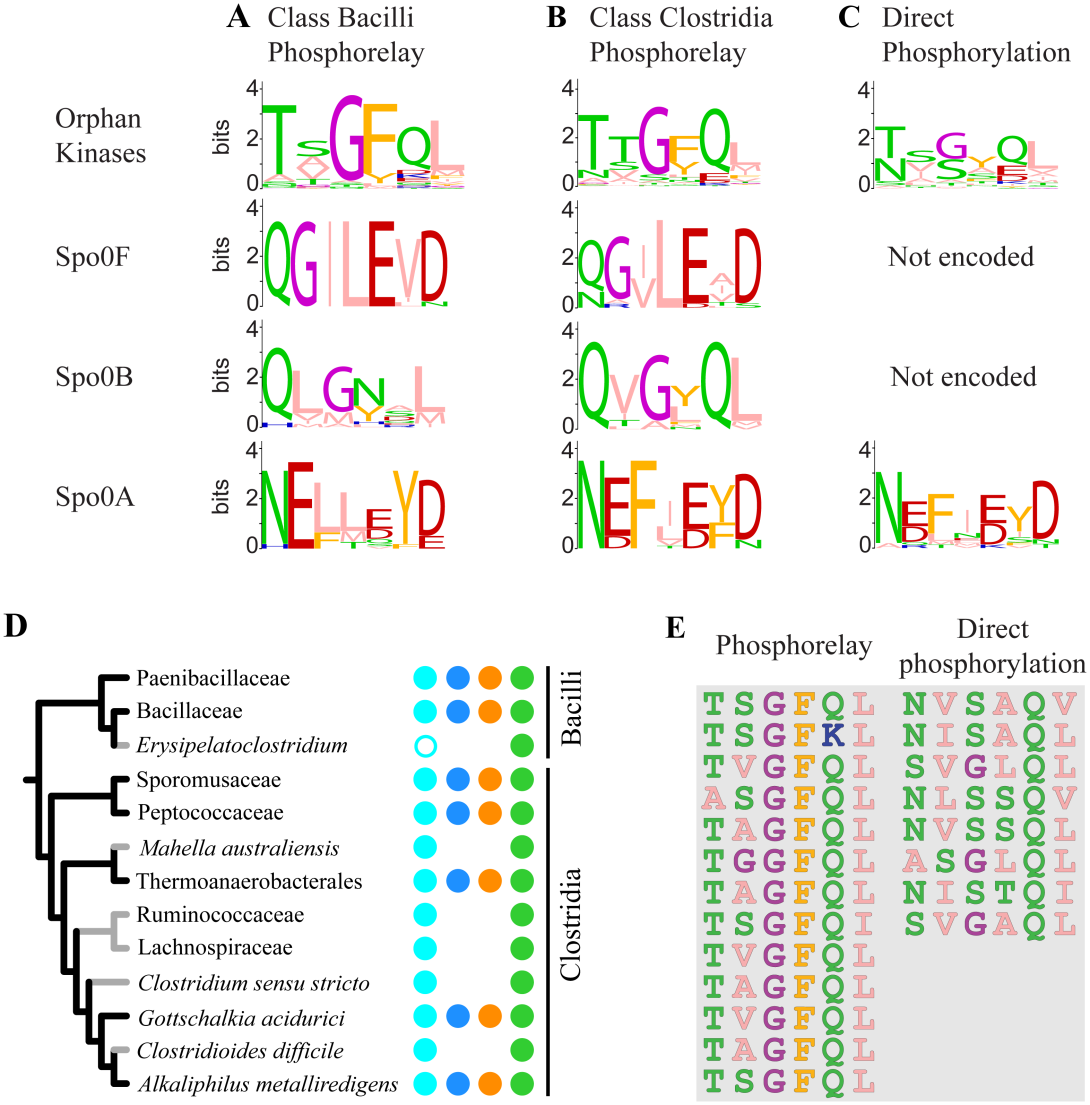
Specificity residues in predicted Spo0 architectures. Sequence logos for predicted specificity residues of orphan kinases, Spo0F, Spo0B, and Spo0A in **(A)** Bacillar phosphorelays, **(B)** Clostridial phosphorelays, and **(C)** direct phosphorylation architectures. Created using WebLogo [72]. **(D)** Clade level summary of the phylogenetic distribution of predicted Spo0 proteins and architectures in spore-forming Firmicutes. Only spore-forming clades shown. Colored branches indicate predicted Spo0 phosphorelay (black) or direct phosphorylation (grey) architecture. Colored circles as in Fig 3 **(E)** Specificity residues of sporulation kinases, experimentally verified in this or prior studies (see also S1 Table), grouped by architecture.

The experimental confirmation of our computational predictions in *D*. *acetoxidans* attests to the reliability of our prediction signatures for both Spo0F and Spo0B. This is corroborated by the consistency of the predictions across the complete data set: almost all species either encode both Spo0F and Spo0B or encode neither. Given that a similarity in specificity residues correlates with phosphotransfer capability *in vitro* [4, 7, 52], we further compared the specificity residues of the predicted Spo0F and Spo0B orthologs, and their putative interaction partners, with the specificity residues of their counterparts in experimentally verified pathways. The predicted specificity residues (S5 Table, Materials and methods), represented as logos (Fig 5A and 5B), show strong similarity to specificity residues in experimentally verified phosphorelay proteins (Table 1; S2 Table). This further supports our prediction of Spo0F and Spo0B orthologs and suggests that they function as intermediate proteins in a phosphorelay architecture.

Based on the combined evidence, we predict that the 33 spore-formers that encode orthologs of Spo0F, Spo0B, and Spo0A possess a phosphorelay. The remaining 15 spore-formers, in which no Spo0F or Spo0B were identified, likely possess a direct phosphorylation architecture. All 48 spore-formers in our data set, with the exception of *Erysipelatoclostridium ramosum*, possess at least one orphan kinase. Of those, 41 have at least one orphan kinase with an N-terminal PAS domain. The predicted pathway architectures agree with the experimental evidence in all species in which the Spo0 pathway architecture has been investigated [13, 16–18, 24, 30, 31].

**Fig 6:**
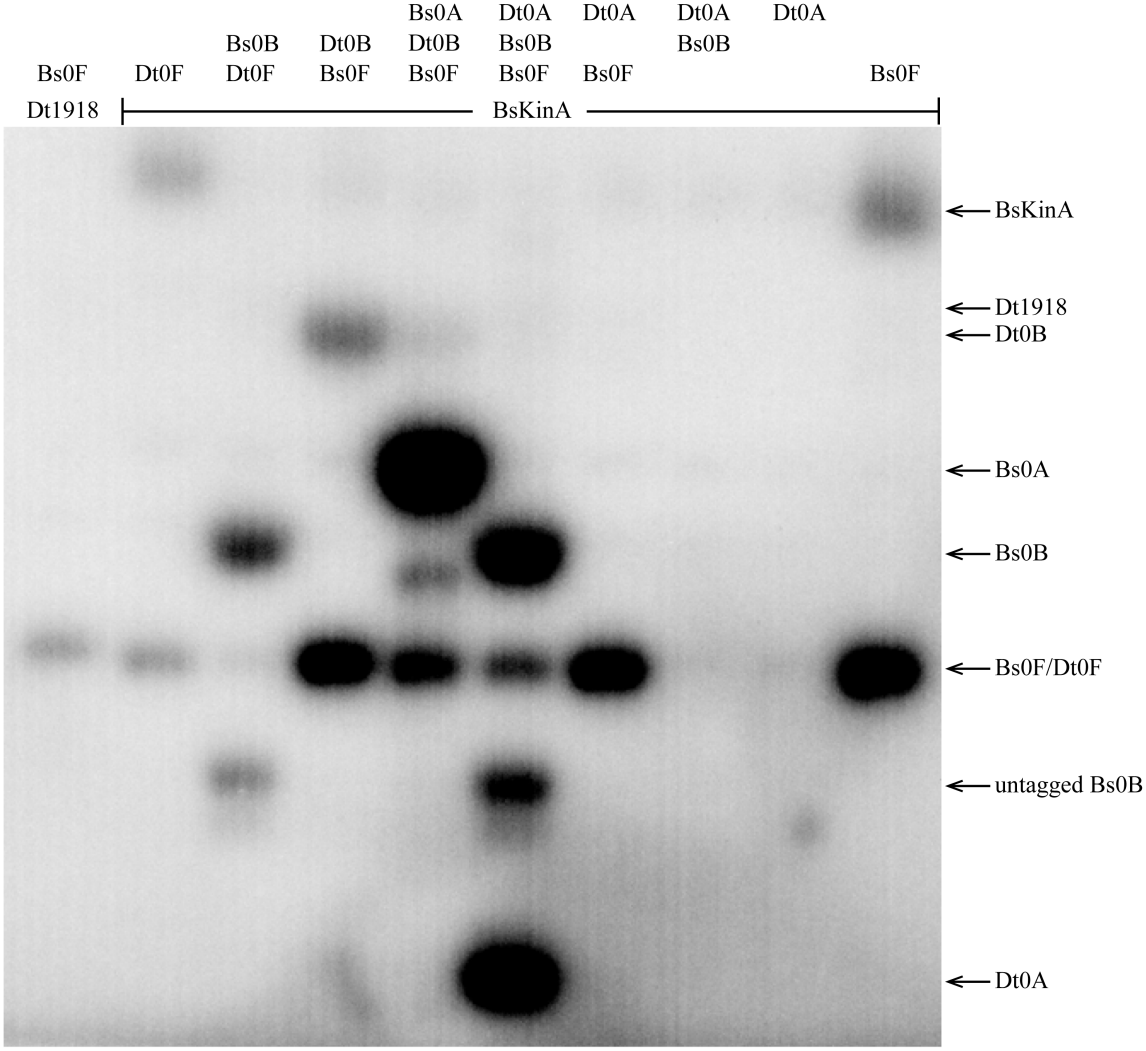
Cross-species complementation of *B*. *subtilis* Spo0 phosphorelay with *D*. *acetoxidans* Spo0 proteins. Phosphotransfer was examined at each transition in the *B*. *subtilis* phosphorelay by systematic replacement of each *B*. *subtilis* protein with its *D*. *acetoxidans* counterpart (lanes 1–6). For comparison, phosphotransfer was also examined in ensembles of proteins lacking one or more constituents of the phosphorelay (lanes 7–10). Consistent with evolutionary conservation of phosphorelay interaction specificity, the *D*. *acetoxidans* phosphorelay protein rescued the phosphodonor and phosphoreceiver functions of the corresponding *B*. *subtilis* phosphorelay protein in each reaction. Phosphorylation of *D*. *acetoxidans* Spo0A by *B*. *subtilis* KinA was not observed, except in the presence of both Spo0F and Spo0B. In each reaction, following autophosphorylation, all proteins combined were incubated for 5 minutes (see text for details). Note that two bands were observed for Bs0B, the lower band likely corresponds to Bs0B that has lost its affinity tag. See Table 2 for abbreviations.

Examination of the phylogenetic distribution of these predicted pathways reveals abundant phosphorelays, not only in Class Bacilli, but also in Class Clostridia. Further, comparison of the specificity residues in phosphorelays predicted in the two classes reveals striking similarity in each Spo0 component (Fig 5A and 5B, see S2 Text for a quantitative comparison), suggesting that the genetic determinants of specificity are similarly encoded in both classes. To test this hypothesis, we asked whether phosphorelay proteins in *D*. *acetoxidans* could recapitulate the function of the corresponding proteins in *B*. *subtilis in vitro* (Table 2, lines 5–8). Specifically, we examined phosphotransfer in the *B*. *subtilis* phosphorelay, as described for *D*. *acetoxidans* above (Fig 4), systematically replacing each *B*. *subtilis* protein with its *D*. *acetoxidans* counterpart (Fig 6, lanes 1–6). For each step in the pathway, a band corresponding to the replacement *D*. *acetoxidans* protein was observed, demonstrating that each *D*. *acetoxidans* protein was capable of accepting a phosphoryl group from the upstream *B*. *subtilis* Spo0 pathway component (Fig 6, lanes 2, 4, and 6). Moreover, bands were observed for downstream components of the *B*. *subtilis* phosphorelay, where included, indicating that the *D*. *acetoxidans* replacement was also capable of transferring a phosphoryl group to the downstream component in the *B*. *subtilis* phosphorelay (Fig 6, lanes 1, 3, 5). Additionally, we noted that phosphorylation of *D*. *acetoxidans* Spo0A by the *B*. *subtilis* phosphorelay required the presence of both *B*. *subtilis* Spo0F and Spo0B proteins (Fig 6, lanes 7–9), indicating that *D*. *acetoxidans* Spo0A cannot be directly phosphorylated by *B*. *subtilis* KinA. Taken together, our results demonstrate that, in all cases, the *D*. *acetoxidans* proteins were able to recapitulate the function of their counterparts in *B*. *subtilis*. Thus, not only do these pathways independently interact as phosphorelays [13, this work], they also encode sufficiently similar phosphotransfer specificity to render them functionally interchangeable, at least *in vitro*. Therefore, either these two phosphorelays arose independently with interchangeable genetic determinants of specificity, which we deem highly unlikely, or these pathways are the descendants of a common ancestral pathway.

### Heterologous interactions reveal changes in kinase specificity across architectures

Having established evidence of common ancestry and conserved specificity in the Spo0 phosphorelay, we next considered the evolutionary history of the direct phosphorylation Spo0 pathway. Examination of the phylogenetic distribution of the predicted architectures in Clostridia (Fig 3, summarized in Fig 5D) reveals that neither predicted architecture is monophyletic. To ensure that this patchiness is not a byproduct of taxon sampling or phylogeny reconstruction artifacts, we repeated the computational analysis with two other Firmicutes phylogenies [43, 44], one of which includes a much larger set of genomes (see S1 Text, S2 and S3 Figs). Both analyses revealed similar patchiness.

**Fig 7:**
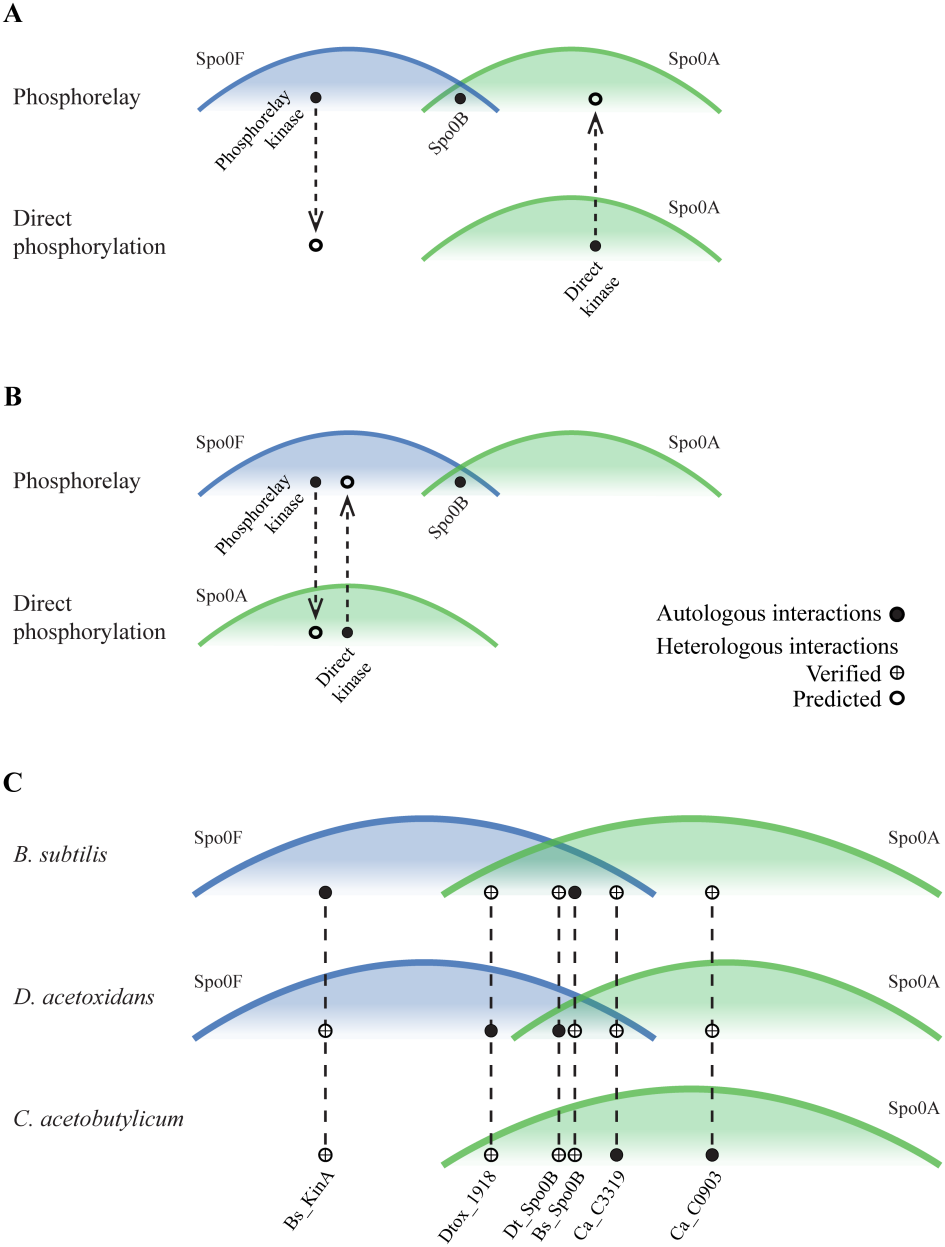
The contributions of change and conservation in Spo0 protein specificity to pathway remodeling. Cross-species phosphotransfer profiling probes the relative positions of Spo0 pathway specificity spectra across architectures. Horizontal axis represents phosphodonor specificity signatures, as in Fig 2. Vertical lines connect points corresponding to the same phosphodonor. **(A)** Schematic showing the positions of receiver specificity spectra in interaction space under the hypothesis that pathway remodeling arose via changes in kinase specificity. The Spo0A specificity spectra are similarly positioned in both architectures. This hypothesis predicts that every kinase that directly phosphorylates Spo0A in its native environment (filled circles) will also be capable of heterologous phosphorylation of Spo0A proteins from phosphorelays (open circles). **(B)** Positions of receiver specificity spectra under the hypothesis that pathway remodeling arose via changes in the Spo0A specificity spectrum. This hypothesis predicts the following heterologous interactions (open circles): Every direct phosphorylation kinase interacts with Spo0F proteins from phosphorelays and every phosphorelay kinase interacts with Spo0A proteins associated with direct phosphorylation architectures. **(C)** Receiver specificity spectra in the *B*. *subtilis* phosphorelay, the *D*. *acetoxidans* phosphorelay, and the *C*. *acetobutylicum* direct phosphorylation architectures. The relative positions of these spectra were inferred from experimentally determined autologous (filled circles) and heterologous (crossed open circles) interactions (summarized in Table 3; see also Figs 6, 8 and 9). The observed interactions support the hypothesis that pathway remodeling arose primarily through changes in kinase specificity, as shown in (A), with a minor shift in the Spo0A specificity spectrum of *D*. *acetoxidans*.

The patchy distribution of predicted Spo0 architectures is consistent with multiple changes in pathway architecture over the course of evolution. Each change in architecture requires the gain of one phosphotransfer interaction and the loss of another. This could occur via changes in kinase specificity residues, resulting in phosphotransfer to a different response regulator (Fig 7A), changes in the Spo0A specificity spectrum, allowing phosphorylation by a different phosphodonor (Fig 7B), or a combination of the two.

To investigate the changes in specificity that resulted in the present-day distribution of Spo0 architectures, we constructed specificity residue logos for orphan kinases and Spo0A proteins encoded in genomes possessing phosphorelays (Fig 5A and 5B), and compared them with the corresponding direct phosphorylation architecture logos (Fig 5C). This comparison revealed that the similarity across architectures is greater for Spo0A proteins than for orphan kinases. When Clostridial and Bacillar phosphorelays are considered separately, Spo0A specificity residues are more similar within the same taxonomic class, than within the same pathway type (Fig 5A–5C; S2 Text, S5 Table). The opposite is true for candidate sporulation kinases. The specificity residues of candidate phosphorelay kinases from both the Clostridia and the Bacilli differ markedly from those of kinases predicted to phosphorylate Spo0A directly. This difference is even more dramatic when experimentally verified sporulation kinases associated with the two architectures are compared (Fig 5E). These results suggest that architectural remodeling was driven primarily by changes in kinase specificity and not changes in Spo0A specificity.

To test this prediction, we probed the heterologous interactions between Spo0 proteins (Table 2) from a Bacillar phosphorelay (*B*. *subtilis*), a Clostridial phosphorelay (*D*. *acetoxidans*), and a Clostridial direct phosphorylation pathway (*C*. *acetobutylicum*). In addition to the phosphorelay kinases, *B*. *subtilis* KinA and *D*. *acetoxidans* Dtox_1918, we included two direct phosphorylation kinases (CA_C0903, CA_C3319) that were chosen to span the diversity of specificity residues observed in experimentally verified sporulation kinases in *C*. *acetobutylicum* [24]. Each of these kinases was incubated with each of the five receiver proteins (two Spo0F and three Spo0A proteins) in separate reactions. To test Spo0B interaction connectivity, each Spo0B protein was incubated with each of the three Spo0A proteins, in separate reactions, in the presence of its autologous kinase and Spo0F.

**Fig 8:**
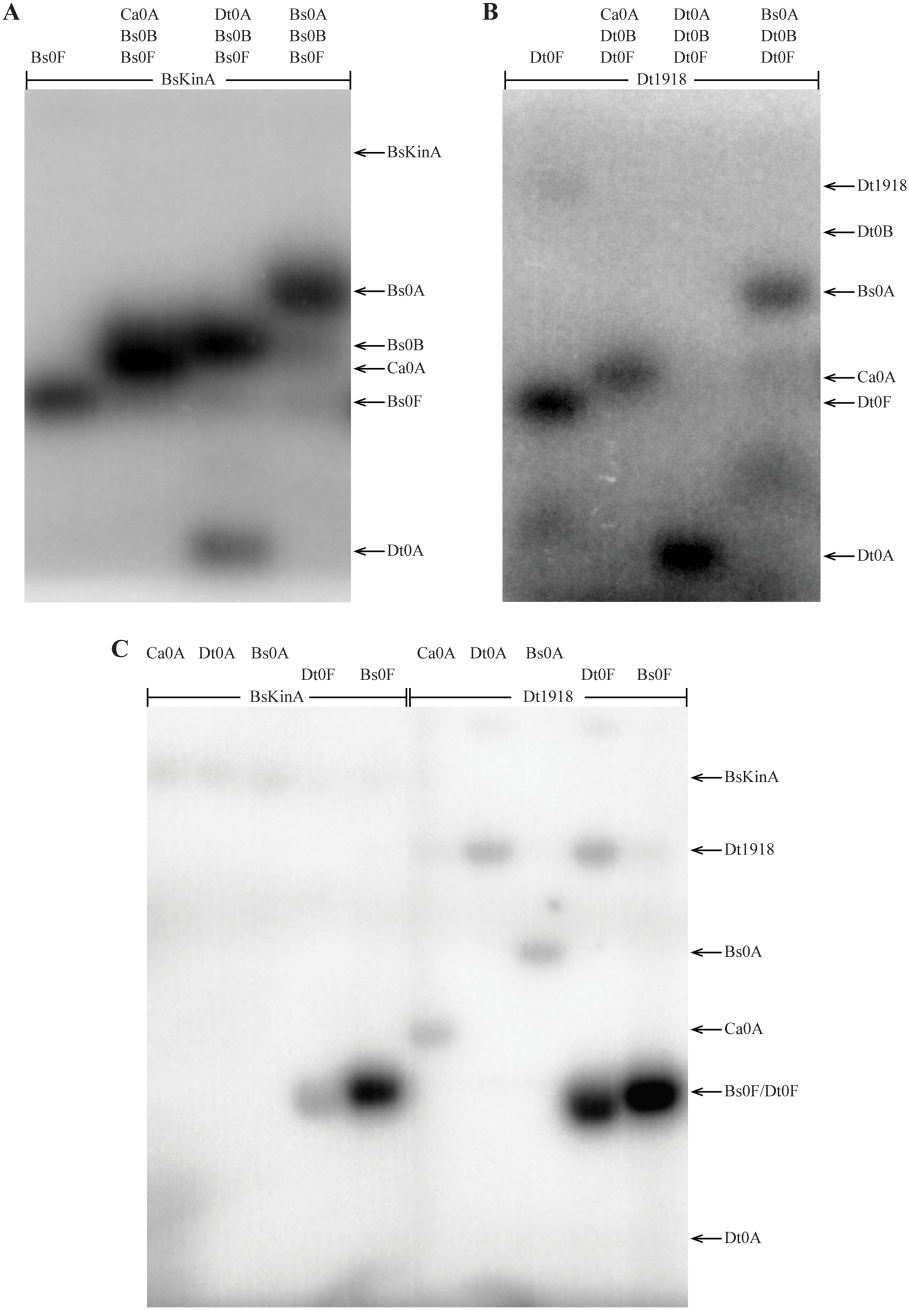
Cross-species phosphotransfer profiling of phosphorelay histidine kinases and Spo0B phosphotransferases. Assessment of phosphotransfer from **(A)** *B*. *subtilis* Spo0B or **(B)** *D*. *acetoxidans* Spo0B to Spo0A proteins from *C*. *acetobutylicum*, *D*. *acetoxidans*, or *B*. *subtilis*, as indicated above each lane. In these reactions, both Bacillar (A) and Clostridial (B) Spo0B specificity residues enable phosphotransfer to Spo0A proteins from both phosphorelays and direct phosphorylation architectures. **(C)** Phosphotransfer was examined from *B*. *subtilis* KinA (lanes 1–5) or *D*. *acetoxidans* Dtox_1918 (lanes 6–10) to Spo0A or Spo0F proteins, as indicated. The observed interactions are consistent with conservation of Spo0A specificity, with minor shifts, across pathway architectures and taxonomic classes. In each reaction, following autophosphorylation, all proteins combined were incubated for 5 minutes (see text for details). See Table 2 for abbreviations.

**Fig 9:**
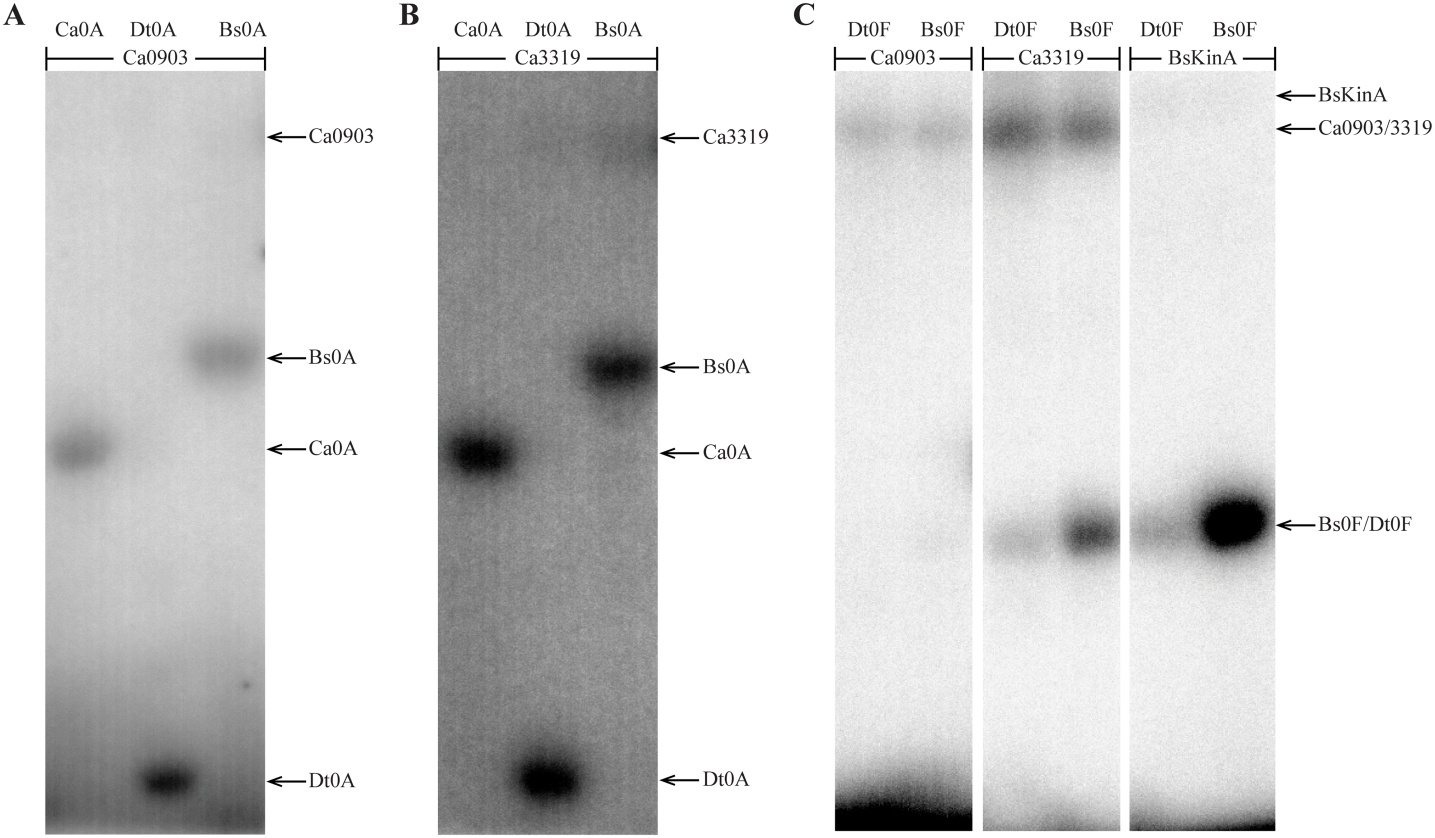
Cross-species phosphotransfer profiling of direct phosphorylation histidine kinases. Assessment of phosphotransfer from *C*. *acetobutylicum* kinases **(A)** Ca_C0903 and **(B)** CA_C3319 to Spo0A proteins from *C*. *acetobutylicum*, *D*. *acetoxidans*, or *B*. *subtilis*, as indicated above each lane. **(C)** Examination of phosphotransfer from Ca_C0903 and CA_C3319 to Spo0F proteins from *D*. *acetoxidans* or *B*. *subtilis*, as indicated. Phosphotransfer to Spo0F proteins by the phosphorelay kinase *B*. *subtilis* KinA, shown for comparison (third panel). The observed interactions are consistent with pathway rewiring via changes in sporulation kinase specificity. In each reaction, following autophosphorylation, all proteins combined were incubated for 5 minutes. See text for details; abbreviations are provided in Table 2.

Interactions between a phosphodonor and a heterologous phosphoreceiver (Figs 8 and 9, summarized in Table 2) allow us to infer the relative positions in interaction space of receiver specificity spectra from various species, because the spectra of receivers that can be phosphorylated by the same donor must overlap. Thus, observation that phosphorelay proteins from *B*. *subtilis* and *D*. *acetoxidans* are functionally interchangeable (Fig 6 and systematically probed in Fig 8) suggests that the specificity spectra of phosphorelay receivers, Spo0F and Spo0A, have changed very little. Moreover, both of the Spo0B proteins tested are capable of phosphorylating Spo0A proteins associated with either phosphorelay or direct phosphorylation architectures (Fig 8A and 8B). Importantly, this shows that the overlapping region of the Spo0F and Spo0A spectra in phosphorelays also overlaps with the specificity spectrum of the directly phosphorylated Spo0A protein in *C*. *acetobutylicum*.

Heterologous interactions also allow us to test hypotheses for the changes in specificity associated with pathway remodeling by probing differences in receiver specificity spectra in phosphorelay and direct phosphorylation architectures. One possibility is that the change in pathway architecture arose through changes in kinase specificity, with little or no change in the Spo0A specificity spectrum. This hypothesis predicts that direct phosphorylation kinases, which phosphorylate Spo0A in their native environments, will also phosphorylate Spo0A proteins from phosphorelays (Fig 7A). An alternate scenario is that the change in pathway architecture is due to changes in Spo0A specificity. This hypothesis predicts that direct phosphorylation kinases will phosphorylate heterologous Spo0F proteins and phosphorelay kinases will phosphorylate Spo0A proteins associated with direct phosphorylation architectures (Fig 7B).

Consistent with the first scenario, both direct phosphorylation kinases tested (CA_C0903, Fig 9A and CA_C3319, Fig 9B) were able to phosphorylate both phosphorelay Spo0A proteins, while only one (CA_C3319, Fig 9C, lanes 3 and 4) was able to phosphorylate Spo0F. In contrast, we only observe two of the four interactions expected in the second scenario: *C*. *acetobutylicum* CA_C0903 did not phosphorylate either of the heterologous Spo0F proteins tested (Fig 9C, lane 1 and 2) and no interaction was observed between *B*. *subtilis* KinA and *C*. *acetobutylicum* Spo0A (Fig 8C, lane 1).

Thus, we observe heterologous interactions that are predicted by the first hypothesis, as well as two additional interactions: First, in addition to both Spo0A proteins, CA_C3319 phosphorylated both Spo0F proteins, indicating that the specificity of CA_C3319 is located in the overlapping region of Spo0F and Spo0A spectra. Selection against crosstalk would act to exclude phosphorelay kinases from this overlapping region; however, such selection would not act on CA_C3319 because Spo0F is not encoded in the same genome. Second, the phosphorelay kinase, Dtox_1918, although unable to phosphorylate the Spo0A encoded in its own genome (Fig 8C, lane 7), phosphorylated both Spo0A proteins encoded in other genomes (Fig 8C, lanes 6 and 8), suggesting a minor shift in the Spo0A spectrum in *D*. *acetobutylicum*, relative to the other species.

The observed heterologous interactions, taken together, support the hypothesis that changes in kinase specificity are the driving force in remodeling of the Spo0 pathway (Table 3, Fig 7C). The requirement that Spo0B must interact with both Spo0F and Spo0A keeps the specificity spectra of those proteins in close proximity in interaction space. Thus, small changes in specificity could easily result in heterologous interactions that would be selectively disadvantageous if they occurred in the native environment, such as those we observe with CA_C3319 and Dtox_1918. The intimate proximity of the Spo0F and Spo0A spectra may contribute to the evolutionary flexibility of the Spo0 phosphorelay.

**Table 3:** Cross-species phosphotransfer interactions.

		Phosphorelay				Direct
		Bs0F	Dt0F	Bs0A	Dt0A	Ca0A
Phosphorelay	BsKinA	+	+	-	-	-
	Bs0B	+	+	+	+	+
	Dt1918	+	+	+	-	+
	Dt0B	+	+	+	+	+
Direct	Ca0903	-	-	+	+	+
	Ca3319	+	+	+	+	+

Summary of cross-species phosphotransfer interactions observed in Figs 6, 8 and 9. Legend: “+”–interaction observed, “-“–no interaction observed.

## Discussion

### The ancestral Spo0 pathway was a phosphorelay

**Fig 10:**
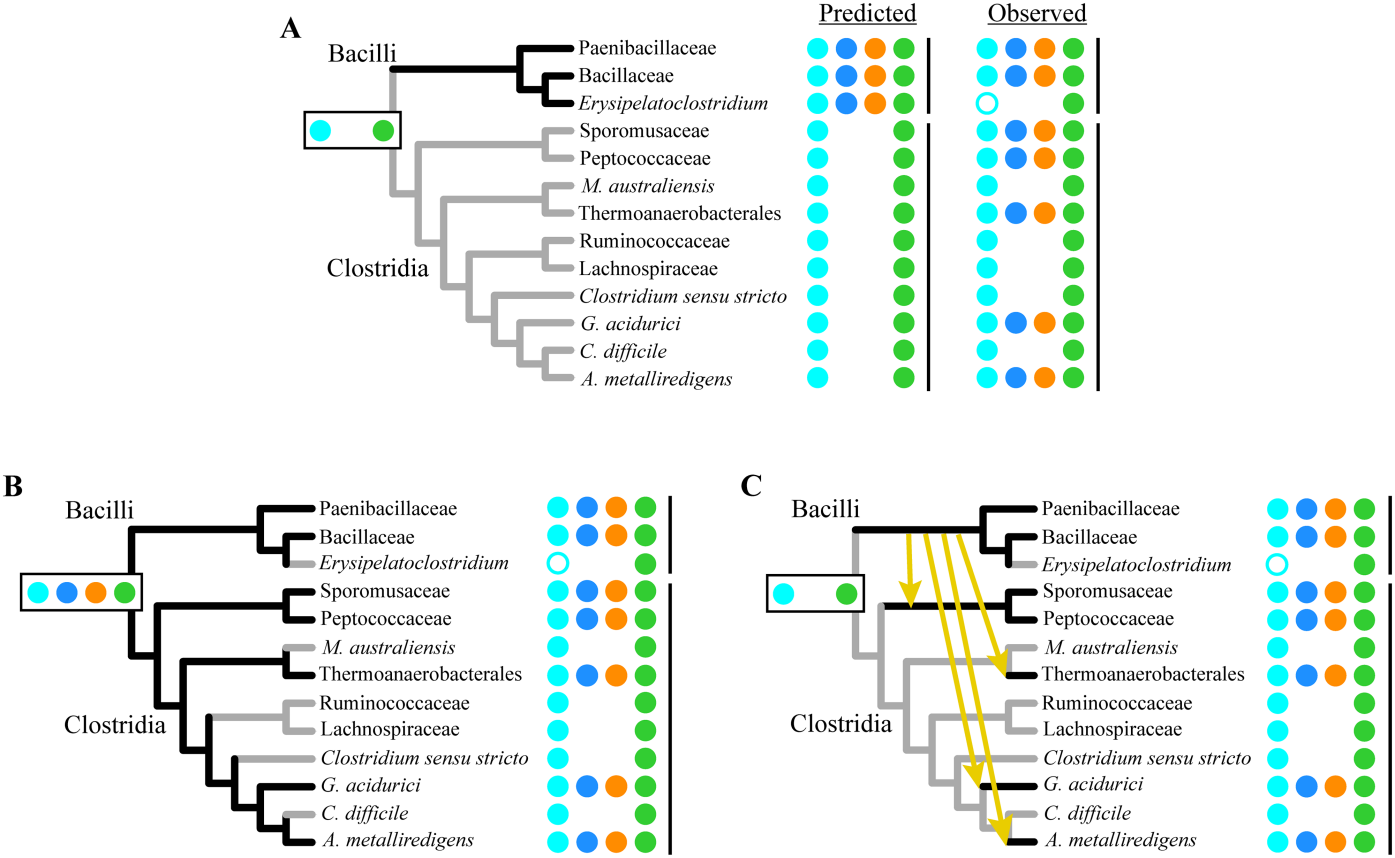
Hypotheses for the evolutionary history of Spo0 architectures. Hypotheses are represented on Firmicute cladograms (only spore-forming clades are shown). Branch color denotes predicted ancestral (internal branches) or extant (leaves) Spo0 pathway architecture (gray: direct phosphorylation architecture; black: phosphorelay). **(A)** The ancestral direct phosphorylation architecture hypothesis [32–34], with emergence of the phosphorelay in the Bacillar ancestor, predicts that phosphorelays and direct phosphorylation architectures in present-day genomes will be restricted to Bacilli and Clostridia, respectively. This distribution is inconsistent with our findings, shown at right. **(B)** The ancestral phosphorelay hypothesis that we propose entails a single invention of the Spo0 phosphorelay, followed by multiple transitions from phosphorelay to direct phosphorylation architecture. Our results are most consistent with this hypothesis. **(C)** An ancestral direct phosphorylation architecture with the present-day Spo0 pathway distribution that is consistent with our findings. This hypothesis requires multiple independent inventions of a phosphorelay or multiple acquisitions of a phosphorelay by horizontal transfer (yellow arrows). We consider both of these scenarios to be unlikely (see text for details).

The juxtaposition of two distinct architectures controlling a homologous sporulation program in anciently related species raises intriguing questions about the role of pathway remodeling during the evolution of the Spo0 pathway. The prevailing hypothesis is that the ancestral Spo0 pathway was a conventional two-component pathway with the emergence of the Bacillar phosphorelay (Fig 10A) following the separation of the classes, Bacilli and Clostridia. This model predicts that phosphorelays will be observed only in Class Bacilli and direct phosphorylation architecture pathways only in Class Clostridia. Our results challenge this model. Using evidence from the conservation of genomic neighborhoods, we identified homologs of the phosphorelay-specific proteins, Spo0F and Spo0B, in several independent lineages in Class Clostridia. Homologs of Spo0F and Spo0B were also found in all spore-formers in Class Bacilli, except two *Erysipelatoclostridium* strains that may be in the process of losing the sporulation phenotype (see S1 Text, Section 3). This patchy distribution calls for a reconsideration of the evolutionary history of the sporulation pathway in the Firmicutes.

We hypothesize that the ancestral Spo0 pathway was a phosphorelay (Fig 10B) and is the ancestor by vertical descent of all present day Spo0 phosphorelays. The sole genesis of the phosphorelay occurred prior to the divergence of Class Bacilli and Class Clostridia. In this scenario, the present-day direct phosphorylation architectures arose through multiple, independent episodes of pathway remodeling, resulting in a patchy distribution of pathway architectures. Whereas multiple independent inventions of a phosphorelay would also result in a patchy distribution, the complexity of the pathway, coupled with the dramatic similarities between predicted phosphorelay proteins from the two classes, render multiple independent inventions of Spo0 phosphorelays unlikely. Further, when we replaced *B*. *subtilis* phosphorelay proteins with their *D*. *acetoxidans* counterparts (Fig 6), every component of the Clostridial phosphorelay was able to recapitulate the interactions of the Bacillar phosphorelay such that connectivity was maintained. It is unlikely that these two pathways encode similar specificity by chance; therefore, we conclude that specificity in the Spo0 phosphorelay has been preserved over 2.7 billion years of independent evolution.

If, as predicted by the standing hypothesis, the phosphorelay first arose in a Bacillar ancestor after the divergence of the Bacilli and the Clostridia (Fig 10C), the present-day distribution of pathway architectures could only occur through horizontal transfer of the phosphorelay to Clostridial taxa. However, acquisition of the phosphorelay through horizontal gene transfer entails an improbable series of events. Multiple independent acquisitions through transfer would be required to produce the present-day distribution, because genomes that harbor a phosphorelay are not monophyletic in the Clostridia. Moreover, each acquisition of the phosphorelay would likely require multiple, independent horizontal transfer events, because the genes encoding Spo0 components are dispersed throughout the genome (S6 Fig). Further, because the genomic neighborhoods of Spo0F and Spo0B are conserved, this scenario requires that every transfer of a gene encoding one of these proteins result in insertion into the same neighborhood. Thus, we conclude that the phosphorelay was most likely present in the ancestor of all Firmicutes and all present-day phosphorelays are derived from it by vertical descent (Fig 10B).

### Remodeling of the Spo0 pathway by changes in sensor kinases

According to the ancestral phosphorelay hypothesis, present-day direct phosphorylation pathways are a result of multiple, independent transitions, wherein Spo0F and Spo0B were lost and direct phosphorylation of Spo0A was gained. Our results support a scenario in which these transitions arose through changes in or replacement of the kinases. Similarities in the genetic determinants of Spo0A specificity reflect shared taxonomic relationships, not shared pathway architecture, consistent with conservation of the Spo0A specificity spectrum throughout the phylum (Fig 5, see also S2 Text). Kinase specificity residues, in contrast, are most similar within the same architecture, consistent with the hypothesis that changes to sensor kinase specificity, and not Spo0A specificity, are responsible for the change in pathway architecture. Further, phosphorelay and direct phosphorylation kinases harbor HK_CA:3 and HK_CA:2 type catalytic domains, respectively (see S3 Text, S6 Table), suggesting that most phosphorelay orphan kinases are distantly related to those of direct phosphorylation architectures. The heterologous phosphotransfer assays (Figs 6–8) also support a history of remodeling through changes in sporulation kinases, and not Spo0A (shown schematically in Fig 10).

### Mechanisms of pathway remodeling

**Fig 11:**
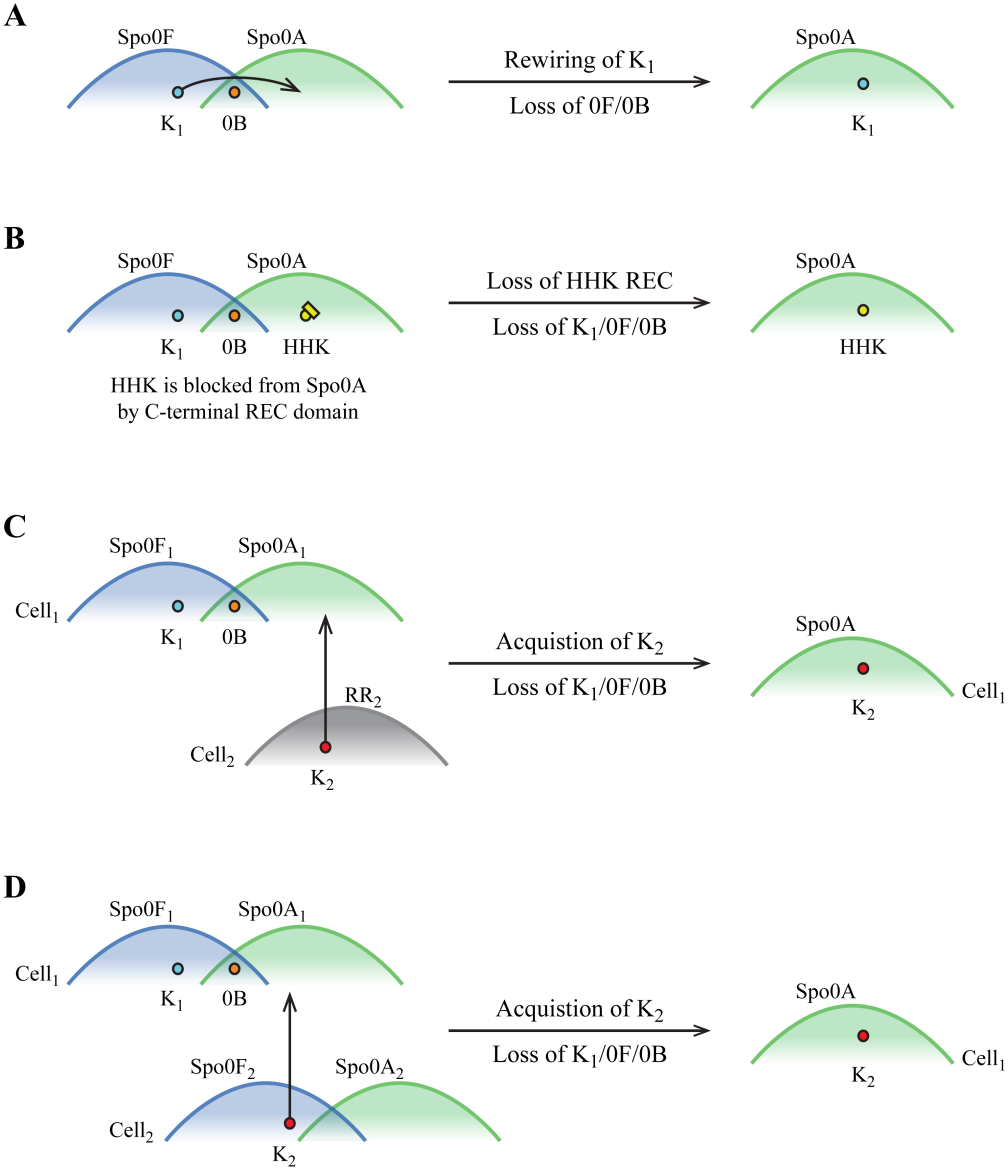
Evolutionary remodeling of a phosphorelay to a direct phosphorylation architecture. Candidate scenarios whereby direct phosphorylation of Spo0A is gained and phosphorelay interactions are lost. **(A)** Sporulation kinase K_1_ accrues substitutions, resulting in a change of specificity from Spo0F to Spo0A. Subsequently, Spo0F and Spo0B are lost. **(B)** A hybrid histidine kinase (HHK) harbors a kinase interaction domain that is specific for Spo0A, but interaction with Spo0A is blocked by its native REC domain [53]. Loss of this REC domain allows interaction with Spo0A. Subsequently, K_1_, Spo0F, and Spo0B are lost. **(C)** Cell_1_ harbors a Spo0 pathway; Cell_2_ does not. Cell_2_ possesses a kinase, K_2_, that is specific for the Spo0A in Cell_1_. Acquisition of K_2_ by horizontal gene transfer results in direct phosphorylation of Spo0A. Subsequently, K_1_, Spo0F, and Spo0B are lost. **(D)** Cell_1_ and Cell_2_ both harbor Spo0 pathways. The specificity spectra for the Spo0 phosphorelay of Cell_2_ are shifted relative to the Spo0 spectra of Cell_1_, such that sporulation kinase K_2_ is specific for the Spo0A in Cell_1_. Acquisition of K_2_ by horizontal gene transfer results in direct phosphorylation of Spo0A. Subsequently, K_1_, Spo0F, and Spo0B are lost.

Here, we highlight several scenarios in which changes in sporulation kinase specificity could result in acquisition of direct phosphorylation of Spo0A. More complex scenarios, for example, involving the interactions between multiple kinases, can also be envisioned. One possibility is that substitutions in an autologous sporulation kinase resulted in a loss of specificity for Spo0F and gain of specificity for Spo0A (Fig 11A). Given the requirement that Spo0F and Spo0A specificity spectra must overlap (since both receivers interact with Spo0B), only a few substitutions may be required. Alternatively, an autologous hybrid histidine kinase, consisting of fused kinase and REC domains, could encode a HisKA domain with pre-existing specificity for Spo0A (Fig 11B), as the REC domain of a hybrid kinase insulates it from interaction with non-cognate receivers [53]. Loss of the REC domain would result in direct phosphorylation of Spo0A.

Acquisition, via horizontal transfer, of a novel kinase already possessing specificity for Spo0A would result in immediate remodeling to a direct phosphorylation architecture. This scenario requires that a kinase encoded in a different species be able to phosphorylate the local Spo0A. This could occur if the donor were a non-sporulating species, in which the specificity spectra associated with Spo0A in spore-formers were occupied by the receiver from an unrelated pathway (Fig 11C). The donor could also be a spore-former if the transferred kinase was insulated from Spo0A in its own cell, but within the Spo0A specificity spectrum of the recipient (Fig 11D), due to minor shifts in the specificity spectra. The interactions observed between *D*. *acetoxidans* Dtox_1918 and heterologous Spo0A proteins suggest that such shifts in the specificity spectra of Spo0F and Spo0A do occur.

The *C*. *acetobutylicum* sporulation kinase, CA_C3319, which exhibited affinity for both Spo0A and Spo0F proteins (Fig 9) may be an example of this last scenario. CA_C3319 harbors a HK_CA:3 type catalytic domain (Agfam [54], see S3 Text), which is commonly observed in phosphorelay sporulation kinases, but not those that directly phosphorylate Spo0A. Further, it possesses unusual specificity residues (SVGLQL) that do not match the typical signatures of either architecture (Table 1; S2 Table). These distinct characteristics suggest that CA_C3319 could be a recently acquired phosphorelay kinase that was specific for Spo0F in the donor cell. Upon acquisition, it may have interacted weakly with Spo0A initially, as there is no Spo0F present in *C*. *acetobutylicum*, and subsequently evolved a stronger preference for Spo0A.

### Repeated, independent remodeling of the Spo pathway

Our results suggest an evolutionary history wherein remodeling of an ancient phosphorelay resulted in a simpler, direct phosphorylation signal transduction pathway. This is consistent with recent theories of reductive genome evolution, which posit that present-day species with streamlined genomes evolve from gene-rich ancestors via a process of specialization [55, 56]. The observation of repeated, independent episodes of pathway remodeling may indicate that the Spo0 pathway has a particular susceptibility for this type of reorganization.

The propensity for pathway remodeling may result from juxtaposition of the particular interaction requirements of the Spo0 phosphorelay and the ecological role of the phenotype that it controls. The specificity spectra of Spo0F and Spo0A must intersect to some extent, since both interact with Spo0B. Given their proximity in interaction space, the mutational trajectories required to lose interaction with Spo0F and gain direct interaction with Spo0A may be short. Further, since sporulation is only essential in survival conditions, selection acting on these mutational trajectories may be relatively permissive. Thus, pathway remodeling via substitutions that change interaction specificity may arise easily.

A second mechanism of pathway remodeling, by acquisition of a foreign kinase with specificity for Spo0A, may be a byproduct of adaptation to changing environments, since acquisition of novel sensor kinases is a source of novel signal recognition capabilities. The diversity of environmental conditions that induce sporulation in various taxa [57], as well as the diversity of lineage specific sporulation kinase repertoires [35] (see also S2 Table), are both consistent with a process of ongoing, lineage-specific turnover of sporulation kinases. Pathway remodeling via acquisition of novel kinases could also be linked to the loss and recovery of the spore formation phenotype. Sporulation is a metabolically expensive process and is lost frequently in stable conditions [23]. Loss of Spo0F or Spo0B is one scenario that would result in loss of sporulation. If environmental conditions subsequently became less favorable, acquisition of a kinase with specificity to Spo0A would restore sporulation, albeit with a direct phosphorylation architecture. Indeed, several *Clostridium sensu stricto* species, which likely encode a direct phosphorylation pathway, nevertheless possess a Spo0B-like protein (Fig 3; S2 and S3 Figs), as might be expected in this scenario. Further, we observe that clades harboring direct phosphorylation architectures tend to encode a mix of spore-formers and non-spore-formers (Fig 3; S2 and S3 Figs), which is consistent with the hypothesis that Spo0 pathway remodeling is linked to loss of sporulation.

What we have learned about the Spo0 phosphorelay suggests general design principles for signaling pathways in which a single protein must interact with multiple partners and specificity is enforced by molecular recognition. It also provides a perspective on the properties that distinguish the Spo0 phosphorelay from other phosphorelays. Sporulation is initiated by multi-input pathways in which each step in the cascade is encoded in a separate protein, requiring that interaction specificity be controlled entirely by molecular recognition. In most phosphorelays that have been studied, two or more of the four interaction domains are encoded in the same protein, such that interaction specificity is controlled by spatial tethering [53]. Signal transduction based on fused proteins that enforce specificity via spatial tethering may be more robust, but less easily reconfigured or expanded. The differences between spatial tethering and molecular recognition in a phosphorelay could represent different trade-offs between flexibility and constraint.

## Materials and methods

### Phylogenetic analysis

To generate a species phylogeny representative of the Firmicutes phylum, we constructed a maximum likelihood tree using 50 concatenated ribosomal protein sequences from 84 Firmicutes genomes, broadly sampled from the major taxonomic families of the Firmicutes phylum. An initial set of aligned ribosomal protein sequences was obtained from [44]. Profiles were constructed from these multiple sequence alignments and used as queries in HMMer [58] to find ribosomal protein family members in the full complement of 84 genomes. A multiple sequence alignment for each ribosomal protein family was constructed separately using GUIDANCE2 [59] with MAFFT [60] to construct the underlying multiple sequence alignment. Columns possessing at least 50% gaps or a GUIDANCE alignment score below 92% were trimmed from the alignment.

Next, the 50 trimmed multiple sequence alignments were concatenated into a supergene alignment. After concatenation, TIGER was used to eliminate uninformative sites [61]. TIGER analysis was performed to group sites into ten bins that are predicted to be evolving at similar rates. The most rapidly evolving sites (Bin_10) were removed, along with columns that were less informative than a randomized site (ptp test with defaults, Bin_Disagreement). The maximum likelihood species tree (Fig 3) was built from the resulting alignment using RaxML version 8.2 [62] with the CAT model [63], which accounts for site-specific heterogeneity, and bootstrapped with 100 replicates (bootstrap values greater than 50 shown as branch labels in Fig 3).

### Genome neighborhood discovery

Candidate homologs of Spo0F and Spo0B were identified based on genome neighborhood conservation. Genome neighborhoods were collected from MistDB.com, version 2.2 [64], which supports protein searches based on RefSeq annotation or domain content (Pfam version 26 [65], Agfam version 1 [54]). Loci up and downstream can also be obtained through the MistDB protein interface. For the purposes of homology identification we refer to the genes encoded four adjacent ORFs up- and downstream as a protein’s genome neighborhood.

First, characteristic neighborhood genes for Spo0F were identified from the genome neighborhood of proteins annotated as Spo0F in Refseq, defined as the Spo0F guideset. RefSeq annotations are incomplete and genes within the neighborhood of many of the genes labelled Spo0F lack a RefSeq gene name annotation. Comparison of genome neighborhoods by sequence similarity is preceded by a matching problem (i.e. which genes should be aligned between neighborhoods). Instead, characteristic domain content, which is algorithmically applied and available as a search term through MistDB, can be used to compare genes within a neighborhood. Thus, to identify potential gene markers we analyzed the domain content of genes within the Spo0F guideset neighborhoods and selected the three domains that were the most frequently observed within the neighborhood and least frequently outside of the neighborhood. Genes encoded close to the Spo0F homolog were also favored as this increases the likelihood that the protein will remain in the neighborhood of Spo0F, even in more distantly related species. By these criteria, we identified the following domains as marker domains for Spo0F neighborhoods: F_bp_aldolase (Fructose Bisphosphate Aldolase, PFAM: PF01116), Transaldolase (recently renamed to TAL_FSA in PFAM version 31, PFAM: PF00923), and CTP_Synth_N (N-terminal CTP synthase, PFAM: PF06418).

To identify candidate Spo0F homologs outside of the guideset, MistDB searches were performed to identify loci encoding a marker domain (see S3 Table, S4 Fig). The genome neighborhoods for all marker genes were collected. Each potential Spo0F neighborhood was searched for proteins matching the Spo0F criteria: a protein encoding only a single REC domain, taking up 90% or more of the total protein (as measured by amino acid coverage). In many cases, the marker genes were encoded within the genome neighborhood. If two or more ORFs containing domains of interest were separated by no more than four ORFs (regardless of length of interstitial non-coding regions), they were combined into the same neighborhood and searched for proteins matching Spo0F, as well as for presentation in S3 Table and S4 Fig. S3 Table presents all identified marker genes and lists a locus for Spo0F in the same row if it was identified in the neighborhood of that marker gene neighborhood. No genome encodes more than one Spo0F and no genome neighborhood contained more than one protein matching the characteristics of Spo0F.

A similar procedure was used to identify candidate Spo0B orthologs (see S4 Table, S5 Fig). Analysis of the neighborhoods of proteins annotated as Spo0B in RefSeq resulted in the selection of three marker genes for the Spo0B neighborhood: GTP1_OBG (PFAM: PF01018), a GTPase domain found on a protein called ObgE in *Bacillus subtilis*, and ribosomal proteins L21 (Ribosomal_L21p, PFAM:PF00829) and L27 (Ribosomal_L27, PFAM:PF01016). For retrieval from MistDB version 2.2 (which uses PFAM version 26), candidate Spo0B proteins fit the criteria of no PFAM domains and included a region alignable to the first 50 amino acids of *B*. *subtilis* Spo0B. In most cases, each of the genes that the marker domains were associated with were singletons and encoded in the same neighborhood of four ORFs. Further, all candidate Spo0Bs were identified encoded between the proteins encoding Ribosomal_L27 and GTP1_OBG proteins. If two or more ORFs containing domains of interest were separated by no more than four ORFs (regardless of length of interstitial non-coding regions), they were combined into the same neighborhood for presentation in S4 Table and S5 Fig.

### Prediction of specificity residues

The specificity residues of candidate Spo0B and sporulation kinase sequences were predicted by manual alignment to the HisKA domains of three *Escherichia coli* kinases, EnvZ, RstB, and CpxA, for which the specificity residues have previously been determined [3]. The conserved histidine residue that holds the phosphoryl group was used to anchor the alignment. Note that Spo0B specificity residues are likely equivalent to those of HisKA because the Spo0F-Spo0B interaction was instrumental in uncovering interfacial contact residues [66] (see also PDB:1F51). Similarly, specificity residues for candidate Spo0F and Spo0A sequences were determined by manual alignment to REC domains of response receivers with known specificity residues (OmpR, RstA, and CpxR from *E*. *coli*) [4]. The resulting predicted specificity residues for phosphodonors and receivers are given in S5 Table. The Spo0F sequence in *Solibacillus silvestris* is truncated and was excluded from this analysis.

### Protein expression constructs

Plasmids encoding Spo0 proteins were constructed for subsequent protein purification and phosphotransfer analysis. Oligonucleotides encoding *Desulfotomaculum acetoxidans* DSM 771 (CP001720.1) and *Clostridium acetobutylicum* ATCC 824 (NC_003030.1) Spo0 protein sequences were designed with codon usage and GC content optimized for expression in *E*. *coli* and synthesized by Genewiz Inc. and Thermo Fisher Scientific GeneArt, respectively. Graphical Codon Usage Analyzer [67] and GeneWiz or Thermo Fisher software were used for coding sequence optimization. Native nucleic acid sequences from the *Bacillus subtilis* subsp. *subtilis* str. 168 genome (NC_000964.3) were used for constructs encoding *B*. *subtilis* Spo0 proteins. To increase protein yield and solubility, truncated sequences possessing intact interaction domains were used in three cases: *D*. *acetoxidans* kinase Dtox_1918 (residues 301–535), *C*. *acetobutylicum* kinase CA_C0903 (residues 244–683, as used in a previous study [24]), and *D*. *acetoxidans* Spo0A (Dtox_2041, residues 1–134). The remaining sequences encode the full-length protein (see S7 Table for GenPept accession numbers). The nucleotide sequences used in all expression constructs are provided in supplementary file S1_Sequence.fasta.

Expression plasmids were created using the Gateway (Invitrogen) recombinational cloning system, as previously described by Laub et al. [68]. Briefly, the nucleotide sequences described above were cloned into pENTR/D-TOPO entry vectors using the pENTR Directional TOPO Cloning Kit (Thermo Fisher Scientific). The coding sequences were subsequently transferred to destination vectors using the Gateway LR reaction (Thermo Fisher Scientific), yielding expression plasmids encoding affinity-tagged proteins under control of an IPTG-inducible promoter. Three N-terminal affinity tags were used: a hexahistidine sequence followed by a thrombin protease cleavage site (His_6_-thrombin); a thioredoxin domain followed by a hexahistidine sequence and a TEV cleavage site (TRX-His_6_-TEV); and a hexahistidine sequence, a maltose-binding protein, and a TEV cleavage site (His_6_-MBP-TEV). The thioredoxin and maltose-binding domains aid protein folding and stability, leading to higher yield during protein purification. Table 2 gives the affinity tag used in each construct, as well as the size and molecular weight of the resulting fusion protein. See S4 Text for a detailed description of the N-terminal fusion sequences used. The complete amino acid sequences of the 11 fusion proteins are provided in S2_Sequence.fasta.

### Protein purification

Proteins were expressed and purified as described in Laub *et al*. [68]. Briefly, constructs in the destination vectors were transformed in *E*. *coli* BL21 cells. These cells were grown in LB medium to an OD_600_ of approximately 0.6 at 37 °C. Protein expression was induced by the addition of 300 μM IPTG, after which cells were incubated at 30 °C for 4 hours. Cells were harvested by centrifugation, transferred to lysis buffer (20 mM Tris-HCl, pH 7.9, 0.5 MNaCl, 10% glycerol, 20 mM imidazole, 0.1%Triton X-100, 1 mM PMSF, 1 mg/ml lysozyme), and sonicated. Cleared lysate was obtained by centrifugation at 30,000g for 60 minutes, and was added to 1mL of equilibrated Ni-NTA agarose slurry (Qiagen). Binding was performed at 4°C for 30 minutes. Next, the Ni-NTA agarose slurry was washed twice in wash buffer (20 mM HEPES-KOH, pH 8.0, 0.5M NaCl, 10% glycerol, 20 mM imidazole, 0.1% Triton X-100, 1 mM PMSF). Tagged proteins were eluted from the slurry using an Econo-column (Bio-Rad) with elution buffer (20 mM HEPE-KOH, pH 8.0, 0.5MNaCl, 10% glycerol, 250 mM imidazole). Finally, PD-10 columns were used to exchange the purified protein into HKEG buffer (10 mM HEPES-KOH, pH8.0, 50mM KCl, 10% glycerol, 0.1 mM EDTA, 2mM DTT) and concentrated, as required.

Purified proteins and a Novex Pre-stained Protein Ladder were visualized on a 12% SDS-Page gel (7.5 μL protein, 2.5 μL 4x LDS Loading Buffer) by staining with Colloidal Blue (all products by Thermo Fisher Scientific). Table 2 summarizes the expected sizes for the proteins used in Figs 4, 6, 8 and 9.

### Autophosphorylation and phosphotransfer profiling reactions

Phosphotransfer profiling reactions were assayed following the protocols described in Laub et al. [68]. Autophosphorylation was performed at an estimated final concentration of 5 μM kinase in HKEG buffer supplemented with 5 mM MgCl_2_, 500 μM ATP, and 0.5 μCi/μL [γ^32^P]-ATP from a stock at ~6000C_i_/mmol (Perkin Elmer). Preliminary autophosphorylation experiments with Dtox_1918 demonstrated that peak autophosphorylation is achieved within 15 minutes and is maintained for at least 30 additional minutes (S7 Fig). We further verified that *D*. *acetoxidans* Spo0B phosphorylation is only observed when both a sporulation kinase and Spo0F are present, suggesting that it does not undergo autophosphorylation (Fig 4B, lane 3, and S8 Fig).

For phosphotransfer profiling of the candidate *D*. *acetoxidans* phosphorelay (Fig 4), Dtox_1918 was incubated with [γ^32^P]-ATP for 15 minutes to allow autophosphorylation. Next, a solution containing radiolabeled Dtox_1918 was split to accommodate three different series of component addition: 1) addition of Spo0F, Spo0B, and Spo0A at 4 minute intervals; 2) addition of Spo0B and Spo0A at 4 minute intervals; 3) addition of Spo0A. A sample was taken 3 minutes after the addition of the next component at each step in the series (Fig 4A). A second sample was taken 10 minutes after the addition of Spo0A in each series (Fig 4B). In all reactions, the estimated final concentrations of the kinase, Spo0F, and Spo0B were 4–6 μM, while the estimated final Spo0A concentration was 10 μM. The reaction for each sample was stopped by the addition of 4X Novex LDS Loading buffer (Life Technologies) and analyzed by 12% SDS-Page gel and phosphorimaging.

For the cross species complementation of the *B*. *subtilis* phosphorelay with *D*. *acetoxidans* phosphorelay components (Fig 6) and the cross-species phosphotransfer profiling experiments (Figs 8 and 9), each kinase was incubated with [γ^32^P]-ATP for 10 minutes to allow autophosphorylation. Next, a mixture of all other Spo0 components was added together to a final volume of 10 μL. The estimated concentration of histidine kinase in the final mixture was 5 μM; all other components had estimated 10 μM concentrations. After a 5 minute incubation, the reaction for each sample was stopped by the addition of SDS-PAGE loading buffer (500 mM Tris-HCl pH 6.8, 8% SDS, 40% glycerol, 400 mM mercaptoethanol) [68] and analyzed by 12% SDS-Page gel and phosphorimaging.

## Supporting information

S1 Table Genomes used in this study. (PDF)

S2 Table Spo0 pathway proteins experimentally verified in prior studies. (XLSX)

S3 Table Spo0F marker gene neighborhoods. (XLSX)

S4 Table Spo0B marker gene neighborhoods. (XLSX)

S5 Table Specificity residues of predicted Spo0 Proteins. (XLSX)

S6 Table Orphan kinase catalytic domain content. (XLSX)

S7 Table Strains and plasmids. (XLSX)

S8 Table Genomic Location of Spo0 Proteins. (XLSX)

S1 Fig Phylogram of 84 representative Firmicute species. Phylogram constructed from the concatenated alignment of 50 ribosomal protein sequences from 84 genomes using RaxML [62], as described in Methods. Outgroup rooted with *Leptotrichia buccalis*. Branch labels represent bootstrap replicates; branch lengths in units of substitutions per site. Colored branches indicate species that are known to sporulate in Class Bacilli (blue) and Class Clostridia (red) (see also S1 Table). Species in which sporulation has not been reported are shown in grey.
(EPS)

S2 Fig Phylogenetic distribution of predicted Spo0 pathway proteins in the Antunes tree. Cladogram of 205 Firmicutes genomes, rooted by 13 outgroup species, adapted from Fig 2 in Antunes *et al*. [43]. Leaves are labeled with the taxonomic names used in the original publication. The names of species that have been recently reclassified, or are under consideration for reclassification, may differ from the nomenclature used in Fig 3 and S1 Fig. Collapsed clades (Veillonellaceae and Lactobacillaceae) represent species that are considered to be non-sporulators [69, 70] and that do not harbor Spo0A, Spo0F, or Spo0B [this work]. The sporulation status of species in other clades is not indicated in this tree. Colored dots indicate Spo0 pathway proteins predicted by the methods used in this study: one or more orphan kinases (cyan); Spo0F (blue); Spo0B (orange); Spo0A (green). Filled cyan dot indicates that at least one orphan kinase encodes a PAS domain.
(EPS)

S3 Fig Phylogenetic distribution of predicted Spo0 pathway proteins in the Yutin tree. Cladogram of 68 Firmicutes genomes, outgroup rooted using *Leptotrichia buccalis* and *Fusibacterium nucleatum*, adapted from Fig 1 in Yutin and Galperin [44]. Leaves are labeled with the taxonomic names used in the original publication. The names of species that have been recently reclassified, or are under consideration for reclassification, may differ from the nomenclature used in Fig 3 and S1 Fig. Putative non-sporulators are shown in grey. Colored dots indicate Spo0 pathway proteins predicted by the methods used in this study: one or more orphan kinases (cyan); Spo0F (blue); Spo0B (orange); Spo0A (green). Filled cyan dots indicates that at least one orphan kinase encodes a PAS domain.
(EPS)

S4 Fig Genome content conservation in regions flanking Spo0F marker genes. Firmicutes cladogram from Fig 3, annotated with Spo0F marker gene neighborhoods. The marker gene neighborhood in each genome was identified as follows: The gene identifiers and domain annotations of the eight genes flanking each Spo0F marker gene (Fructose bisphosphate aldolase, Transaldolase, and CTP Synthase) were retrieved from MistDB [64]. Sets of genes with at least one gene in common were combined, resulting in one to three non-overlapping sets of contiguous genes. The Spo0F marker neighborhood is defined to be the set of genes that contains a spo0F-like gene (i.e., a gene encoding a stand-alone REC domain). If none contains a spo0F-like gene, then the set that contains the most marker genes is selected, if one exists. Otherwise, one of the three sets is chosen arbitrarily. Note that *Peptostreptococcus anaerobius* and *Mageeibacillus indolicus* lack marker gene neighborhoods because no annotated Spo0F marker genes were found in these genomes. Spo0F marker gene neighborhoods are displayed with the homologous families that occur most frequently in those neighborhoods indicated in color (see legend); the remaining genes shown in white. Homologous genes were identified based on shared domain content. Colored branches indicate species that are known to sporulate in Class Bacilli (blue) and Class Clostridia (red) (see also S1 Table). Species in which sporulation has not been reported are shown in grey.
(EPS)

S5 Fig Genome content conservation in regions flanking Spo0B marker genes. Firmicutes cladogram from Fig 3, annotated with Spo0B marker gene neighborhoods. The marker gene neighborhood in each genome was identified as follows: The gene identifiers and domain annotations of the eight genes flanking each Spo0B marker gene (L21, L27, and ObgE) were retrieved from MistDB [64]. Sets of genes with at least one gene in common were combined, resulting in one to three non-overlapping sets of contiguous genes. The Spo0B marker neighborhood is defined to be the set of genes that contains a spo0B-like gene (as described in the main text). If none contains a spo0B-like gene, then the set that contains the most marker genes is selected, if one exists. Otherwise, a set is chosen arbitrarily. Spo0B marker gene neighborhoods displayed with the homologous families that occur most frequently in those neighborhoods indicated in color (see legend); the remaining ORFs shown in white. Homologous genes were identified based on shared domain content. Colored branches indicate species that are known to sporulate in Class Bacilli (blue) and Class Clostridia (red) (see also S1 Table). Species in which sporulation has not been reported are shown in grey.
(EPS)

S6 Fig Spatial distribution of Spo0-encoding genes along the genome. Genomic location of genes encoding predicted Spo0 proteins in **(A)** *B*. *subtilis* (5 orphan kinases) and **(B)** all spore-forming members of the representative Firmicutes used in this study for which a complete, fully assembled genome sequence is available (40 genomes, 204 orphan kinases). Normalized position calculated based on the position of the start codon (or the stop codon for proteins encoded on the complementary strand), divided by the length of the genome in base pairs (see S8 Table). Orphan histidine kinases, Spo0B, and Spo0A (shown in teal, orange, and green, respectively) are dispersed throughout the genomes; Spo0F (blue) is commonly located near the origin of replication. Separation by **(C)** taxonomic class (Bacilli:14 genomes, 88 orphan kinases; Clostridia: 26 genomes, 116 orphan kinases) or **(D)** predicted Spo0 architecture (phosphorelay: 29 genomes, 158 orphan kinases; direct phosphorylation: 11 genomes, 46 orphan kinases) does not change the observed distribution of orphan kinases, Spo0B, or Spo0A; nor does it affect the localization of Spo0F near the origin.
(EPS)

S7 Fig Time course of Dtox_1918 kinase autophosphorylation. Radiograph of Dtox_1918 time course indicates that peak autophosphorylation is achieved within 15 minutes of addition of radiolabeled ATP. Each lane contains a sample from an incubation of 5 μM kinase in HKEG buffer, supplemented with 5 mM MgCl_2_, 500 μM ATP, and 0.5 μCi/μL [γ^32^P]-ATP from a stock at ~6000C_i_/mmol (Perkin Elmer). Samples were mixed with LDS prior to separation on a 10% SDS-PAGE gel.
(EPS)

S8 Fig Lack of evidence for Spo0B autophosphorylation. Phosphotransfer from Dtox_1918 was assessed in incubations with Dtox_Spo0F, Dtox_Spo0B, or both, as indicated. Reactions were sampled 5, 15, and 30 minutes after initiation of the reaction and mixed with LDS prior to separation on a 10% SDS-PAGE gel. Dtox_Spo0B phosphorylation is only observed in the presence of both Dtox_1918 and Dtox_Spo0F. No Dtox_Spo0B band is observed when incubated with Dtox_1918 alone, indicating that Dtox_1918 does not directly phosphorylate Dtox_Spo0B and that Dtox_Spo0B is does not autophosphorylate under the conditions used in this study. All incubations were carried out in HKEG buffer, supplemented with 5 mM MgCl_2_, 500 μM ATP, and 0.5 μCi/μL [γ^32^P]-ATP from a stock at ~6000C_i_/mmol (Perkin Elmer), with the following estimated protein concentrations: Dtox_1918, 2 μM; Dtox_Spo0F, 7 μM; and Dtox_Spo0B, 5 μM.
(EPS)

S1 Text Comparison of the distribution of predicted Spo0 pathways in three Firmicute phylogenies. (PDF)

S2 Text Quantitative comparison of specificity residues. (PDF)

S3 Text Comparison of orphan kinase catalytic domain content. (PDF)

S4 Text Construction of expression vectors for N-terminal fusion proteins. (PDF)

S1 Sequence Oligonucleotide sequences used in the protein expression constructs. See S4 Text for details.
(FASTA)

S2 Sequence Amino acid sequences of N-terminally tagged Spo0 proteins. See S4 Text for details.
(FASTA)
