# Supplementary material for: Flexibility and constraint: Evolutionary remodeling of the sporulation initiation pathway in Firmicutes
Source: PLoS Genet. 2018 Sep 13;14(9):e1007470. doi: 10.1371/journal.pgen.1007470 (PMC6136694; doi:10.1371/journal.pgen.1007470)
Supplement: S1 Table — (PDF) [file pgen.1007470.s001.pdf]

S1 Table. Genomes used in this study.

Species Name	Sporulates	Accession	Source[1]
Acetivibrio cellulolyticus CD2	No	JH556651	Patel et al. [1980]
Acetonema longum DSM 6540	Yes	AFGF01000013	Tocheva et al. [2011]
Acholeplasma laidlawii PG-8A	No	NC_010163	Vishniakov et al. [2015]
Acidaminococcus fermentans DSM 20731	No	NC_013740	Chang et al. [2010]
Alicyclobacillus acidocaldarius subsp. *acidocaldarius* DSM 446	Yes	NC_013208	Palop et al. [2000]
Alkaliphilus metalliredigens QYMF	Yes	NC_009633	Ye et al. [2004]
Ammonifex degensii KC4	No	NC_013386	Huber et al. [1996]
Anoxybacillus flavithermus WK1	Yes	NC_011567	Pikuta et al. [2000]
Bacillus anthracis str. Ames	Yes	MF101005	
Bacillus clausii KSM-K16	Yes	NC_006582	
Bacillus halodurans C-125	Yes	BA000004	
Bacillus megaterium DSM 319	Yes	HG781834	Eppinger et al. [2011]
Bacillus psychrosaccharolyticus ATCC 23296	Yes	NZ_AJTN00000000	Seo et al. [2004]
Bacillus subtilis subsp. *subtilis* str. 168	Yes	NC_000964	Nakamura et al. [1999]
Blautia hansenii DSM 20583	No	NZ_CP022413	Liu et al. [2008]
Brevibacillus brevis NBRC 100599	Yes	NC_012491	Shida et al. [1996]
Butyrivibrio proteoclasticus B316	No	NC_014388	Moon et al. [2008]
Caldicellulosiruptor saccharolyticus DSM 8903	No	NC_009437	Willquist et al. [2010]
Candidatus Arthromitus sp. *SFB-mouse-Yit*	Yes	NC_017294	Thompson et al. [2012]

S1 Table. (continued)

Species Name	Sporulates	Accession	Source[1]
Candidatus Desulforudis audaxviator MP104C	Yes	NC_010424	Chivian et al. [2008]
Carboxydothermus hydrogenoformans Z-2901	Yes	NC_007503	Wu et al. [2005]
Centipeda periodontii DSM 2778	No	NZ_AFHQ00000000	Lai et al. [1983]
Clostridium acetobutylicum ATCC 824	Yes	NC_003030	Steiner et al. [2011]
Clostridium botulinum A str. Hall	Yes	NC_009698	Lindström and Korkeala [2006]
Clostridium lentocellum DSM 5427	Yes	NC_015275	Miller et al. [2011]
Clostridium perfringens ATCC 13124	Yes	NC_008261	Paredes-Sabja et al. [2008]
Clostridium tetani E88	Yes	NC_004565	Bruggemann et al. [2003]
Desulfosporosinus orientis DSM 765	Yes	NC_016584	Robertson et al. [2000]
Desulfotomaculum acetoxidans DSM 771	Yes	CP001720	Widdel and Pfennig [1977]
Desulfotomaculum reducens MI-1	Yes	CP000612	Junier et al. [2009]
Desulfotomaculum ruminis DSM 2154	Yes	NC_015589	Spring et al. [2012]
Dorea formicigenerans ATCC 27755	No	NZ_AAXA00000000	Taras et al. [2002]
Enterococcus faecalis V583	No	NC_004668	Naser et al. [2005]
Erysipelatoclostridium ramosum DSM 1402	Yes	NZ_ABFX00000000	Yutin and Galperin [2013]
Erysipelatoclostridium spiroforme DSM 1552	Yes	NZ_ABIK00000000	Yutin and Galperin [2013]
Erysipelothrix rhusiopathiae str. Fujisawa	No	NC_015601	Soriano et al. [1998]
Ethanoligenens harbinense YUAN-3	No	NC_014828	Xing et al. [2006]
Eubacterium eligens ATCC 27750	No	NC_012778	
Eubacterium rectale ATCC 33656	No	NC_012781	

S1 Table. (continued)

Species Name	Sporulates	Accession	Source[1]
Exiguobacterium sibiricum 255-15	No	NC_010556	Tena et al. [2014]
Filifactor alocis ATCC 35896	No	CP002390	Jalava and Eerola [1999]
Finegoldia magna ATCC 29328	No	NC_010376	Murdoch and Shah [1999]
Geobacillus kaustophilus HTA426	Yes	NC_006510	Nazina et al. [2001]
Gottschalkia acidurici 9a	Yes	CP003326	Hartwich et al. [2012]
Heliobacterium modesticaldum Ice1	Yes	NC_010337	Kimble-Long and Madigan [2001]
Kyrpidia tusciae DSM 2912	Yes	NC_014098	Klenk et al. [2011]
Lachnoclostridium phytofermentans ISDg	Yes	NC_010001	Warnick et al. [2002]
Lachnoclostridium symbiosum WAL-14163	Yes	NZ_ADLQ00000000	Allen et al. [2003]
Lactobacillus casei ATCC 334	No	NC_008526	
Leptotrichia buccalis C-1013-b	No	NC_013192	Ivanova et al. [2009]
Listeria monocytogenes EGD-e	No	NC_003210	Tan et al. [2011]
Lysinibacillus fusiformis ZC1	Yes	NZ_CP010820	Ahmed et al. [2007]
Lysinibacillus sphaericus C3-41	Yes	NZ_CP017560	Ahmed et al. [2007]
Macrococcus caseolyticus JCSC5402	No	NC_011999	Kloos et al. [1998]
Mageeibacillus indolicus UPII9-5	No	NC_013895	Austin et al. [2015]
Mahella australiensis 50-1 BON	Yes	NC_015520	Salinas et al. [2004]
Megasphaera elsdenii DSM 20460	No	NC_015873	Marounek et al. [1989]
Moorella thermoacetica ATCC 39073	Yes	CP000232	Byrer et al. [2000]
Oceanobacillus iheyensis HTE831	Yes	NC_004193	Lu et al. [2001]
Paenibacillus polymyxa E681	Yes	CP000154	Park et al. [2012]
Pelosinus fermentans DSM 17108	Yes	NZ_CP010978	Shelobolina et al. [2007]
Pelotomaculum thermopropionicum SI	Yes	AP009389	Imachi et al. [2002]

S1 Table. (continued)

Species Name	Sporulates	Accession	Source[1]
Clostridioides difficile 630	Yes	NC_009089	Paredes-Sabja et al. [2014]
Peptoclostridium sticklandii DSM 519	No	NC_014614	Stadtman and McClung [1957]
Peptostreptococcus anaerobius VPI 4330	No	NZ_ARMA00000000	Higaki et al. [2000]
Phascolarctobacterium succinatutens YIT 12067	No	NZ_AEVN00000000	Watanabe et al. [2012]
Planococcus antarcticus DSM 14505	No	NZ_CP016534	Reddy et al. [2002]
Roseburia hominis A2-183	No	NC_015977	Stanton and Savage [1983]
Ruminiclostridium cellulolyticum H10	Yes	NC_011898	Petitdemange et al. [1984]
Ruminiclostridium leptum DSM 753	Yes	NZ_ABCB00000000	Moore et al. [1976]
Ruminiclostridium thermocellum ATCC 27405	Yes	NC_009012	Freier et al. [1988]
Ruminococcus albus 7	No	NC_014833	Ntaikou et al. [2008]
Selenomonas ruminantium subsp. *lactilytica* TAM6421	No	NZ_JNIO00000000	Stackebrandt et al. [1985]
Solibacillus silvestris StLB046	Yes	NZ_CP014609	Krishnamurthi et al. [2009]
Staphylococcus aureus subsp. *aureus* NCTC 8325	No	NC_007795	
Streptococcus pneumoniae TIGR4	No	NC_003098	
Syntrophomonas wolfei subsp. *wolfei* str. Goettingen	No	NC_008346	Sieber et al. [2010]
Syntrophothermus lipocalidus DSM 12680	No	NC_014220	Sekiguchi et al. [2000]
Thermincola potens JR	No	NC_014152	Sokolova et al. [2004]
Thermoanaerobacter italicus Ab9	Yes	NC_013921	Kozianowski et al. [1997]

S1 Table. (continued)

Species Name	Sporulates	Accession	Source[1]
Thermoanaerobacter pseudethanolicus ATCC 33223	Yes	NC_010321	Onyenwoke et al. [2007]
Thermoanaerobacterium thermosaccharolyticum DSM 571	Yes	NC_014410	O-Thong et al. [2008]
Thermoanaerobacterium xylanolyticum LX-11	Yes	NC_015555	Lee et al. [1993]
Thermosinus carboxydivorans Nor1	No	NZ_AAWL00000000	Sokolova et al. [2004]
Veillonella parvula DSM 2008	No	NC_013520	Gronow et al. [2010]

[1] Evidence for sporulation status, if known. If no source provided, sporulation status is inferred from the closest classification described in Bergey's Manual of Systematic Bacteriology [Vos et al., 2009]

Bibliography

I. Ahmed, A. Yokota, A. Yamazoe, and T. Fujiwara. Proposal of *Lysinibacillus boronitolerans* gen. nov. sp. nov., and transfer of *Bacillus fusiformis* to *Lysinibacillus fusiformis* comb. nov. and *Bacillus sphaericus* to *Lysinibacillus sphaericus* comb. nov. *Int J Syst Evol Microbiol*, 57: 1117–1125, May 2007.

S. D. Allen, C. L. Emery, and D. M. Lyerly. *Clostridium in Manual of Clinical Microbiology*, pages 835–856. American Society for Microbiology, Washington D.C., 8th edition, 2003.

M. Austin, L. Rabe, S. Srinivasan, D. Fredricks, H. Wiesenfeld, and S. Hillier. *Mageeibacillus indolicus* gen. nov., sp. nov.: a novel bacterium isolated from the female genital tract. *Anaerobe*, 32:37–42, Apr 2015.

H. Bruggemann, S. Baumer, W. Fricke, A. Wiezer, H. Liesegang, I. Decker, C. Herzberg, R. Martinez-Arias, R. Merkl, A. Henne, and G. Gottschalk. The genome sequence of *Clostridium tetani*, the causative agent of tetanus disease. *Proc Natl Acad Sci U S A*, 100:1316–1321, Feb 2003.

D. Byrer, F. Rainey, and J. Wiegel. Novel strains of *Moorella thermoacetica* form unusually heat-resistant spores. *Arch Microbiol*, 174:334–339, Nov 2000.

Y. Chang, R. Pukall, E. Saunders, A. Lapidus, A. Copeland, M. Nolan, T. Glavina Del Rio, S. Lucas, F. Chen, H. Tice, J. Cheng, C. Han, J. Detter, D. Bruce, L. Goodwin, S. Pitluck, N. Mikhailova, K. Liolios, A. Pati, N. Ivanova, K. Mavromatis, A. Chen, K. Palaniappan, M. Land, L. Hauser, C. Jeffries, T. Brettin, M. Rohde, M. Göker, J. Bristow, J. Eisen, V. Markowitz, P. Hugenholtz, N. Kyrpides, and H. Klenk. Complete genome sequence of *Acidaminococcus fermentans* type strain (vr4). *Stand Genomic Sci*, 3:1–14, Jul 2010.

D. Chivian, E. Brodie, E. Alm, D. Culley, P. Dehal, T. DeSantis, T. Gihring, A. Lapidus, L. Lin, S. Lowry, D. Moser, P. Richardson, G. Southam, G. Wanger, L. Pratt, G. Andersen, T. Hazen, F. Brockman, A. Arkin, and T. Onstott. Environmental genomics reveals a single-species ecosystem deep within earth. *Science*, 322:275–278, Oct 2008.

M. Eppinger, B. Bunk, M. Johns, J. Edirisinghe, K. Kutumbaka, S. Koenig, H. Creasy, M. Rosovitz, D. Riley, S. Daugherty, M. Martin, L. Elbourne, I. Paulsen, R. Biedendieck, C. Braun,

S. Grayburn, S. Dhingra, V. Lukyanchuk, B. Ball, R. Ul-Qamar, J. Seibel, E. Bremer, D. Jahn, J. Ravel, and P. Vary. Genome sequences of the biotechnologically important *Bacillus megaterium* strains QM B1551 and DSM319. *J Bacteriol*, 193:4199–4213, Aug 2011.

D. Freier, C. Mothershed, and J. Wiegel. Characterization of *Clostridium thermocellum* JW20. *Appl Environ Microbiol*, 54:204–211, Jan 1988.

S. Gronow, S. Welnitz, A. Lapidus, M. Nolan, N. Ivanova, T. Glavina Del Rio, A. Copeland, F. Chen, H. Tice, S. Pitluck, J. Cheng, E. Saunders, T. Brettin, C. Han, J. Detter, D. Bruce, L. Goodwin, M. Land, L. Hauser, Y. Chang, C. Jeffries, A. Pati, K. Mavromatis, N. Mikhailova, A. Chen, K. Palaniappan, P. Chain, M. Rohde, M. Göker, J. Bristow, J. Eisen, V. Markowitz, P. Hugenholtz, N. Kyrpides, H. Klenk, and S. Lucas. Complete genome sequence of *Veillonella parvula* type strain (Te3). *Stand Genomic Sci*, 2:57–65, Jan 2010.

K. Hartwich, A. Poehlein, and R. Daniel. The purine-utilizing bacterium *Clostridium acidurici* 9a: a genome-guided metabolic reconsideration. *PLoS One*, 7:e51662, Dec 2012.

S. Higaki, T. Kitagawa, M. Kagoura, M. Morohashi, and T. Yamagishi. Characterization of *Peptostreptococcus* species in skin infections. *J Int Med Res*, 28:143–147, May/Jun 2000.

R. Huber, P. Rossnagel, C. R. Woese, R. Rachel, T. A. Langworthy, and K. O. Stetter. Formation of ammonium from nitrate during chemolithoautotrophic growth of the extremely thermophilic bacterium *Ammonifex degensii* gen. nov. sp. nov. *Syst Appl Microbiol*, 19:40–49, Mar 1996.

H. Imachi, Y. Sekiguchi, Y. Kamagata, S. Hanada, A. Ohashi, and H. Harada. *Pelotomaculum thermopropionicum* gen. nov., sp. nov., an anaerobic, thermophilic, syntrophic propionate-oxidizing bacterium. *Int J Syst Evol Microbiol*, 52:1729–1735, Sep 2002.

N. Ivanova, S. Gronow, A. Lapidus, A. Copeland, T. Glavina Del Rio, M. Nolan, S. Lucas, F. Chen, H. Tice, J. Cheng, E. Saunders, D. Bruce, L. Goodwin, T. Brettin, J. Detter, C. Han, S. Pitluck, N. Mikhailova, A. Pati, K. Mavrommatis, A. Chen, K. Palaniappan, M. Land, L. Hauser, Y. Chang, C. Jeffries, P. Chain, C. Rohde, M. Göker, J. Bristow, J. Eisen, V. Markowitz, P. Hugenholtz, N. Kyrpides, and H. Klenk. Complete genome sequence of *Leptotrichia buccalis* type strain (C-1013-b). *Stand Genomic Sci*, 1:126–132, Sep 2009.

J. Jalava and E. Eerola. Phylogenetic analysis of *Fusobacterium alocis* and *Fusobacterium sulci* based on 16S rRNA gene sequences: proposal of *Filifactor alocis* (Cato, Moore and Moore) comb. nov. and *Eubacterium sulci* (Cato, Moore and Moore) comb. nov. *Int J Syst Bacteriol*, 49 Pt 4:1375–1379, Oct 1999.

P. Junier, M. Frutschi, N. Wigginton, E. Schofield, J. Bargar, and R. Bernier-Latmani. Metal reduction by spores of *Desulfotomaculum reducens*. *Environ Microbiol*, 11:3007–3017, Dec 2009.

L. Kimble-Long and M. Madigan. Molecular evidence that the capacity for endosporulation is universal among phototrophic heliobacteria. *FEMS Microbiol Lett*, 199:191–195, May 2001.

H. Klenk, A. Lapidus, O. Chertkov, A. Copeland, T. Del Rio, M. Nolan, S. Lucas, F. Chen, H. Tice, J. Cheng, C. Han, D. Bruce, L. Goodwin, S. Pitluck, A. Pati, N. Ivanova, K. Mavromatis, C. Daum, A. Chen, K. Palaniappan, Y. Chang, M. Land, L. Hauser, C. Jeffries, J. Detter, M. Rohde, B. Abt, R. Pukall, M. Göker, J. Bristow, V. Markowitz, P. Hugenholtz, and J. Eisen. Complete genome sequence of the thermophilic, hydrogen-oxidizing *Bacillus tusciae* type strain (T2) and reclassification in the new genus, *Kyrpidia* gen. nov. as *Kyrpidia tusciae* comb. nov. and emendation of the family Alicyclobacillaceae da Costa and Rainey, 2010. *Stand Genomic Sci*, 5:121–134, Oct 2011.

W. Kloos, D. Ballard, C. George, J. Webster, R. Hubner, W. Ludwig, K. Schleifer, F. Fiedler, and K. Schubert. Delimiting the genus *Staphylococcus* through description of *Macrococcus caseolyticus* gen. nov., comb. nov. and *Macrococcus equipercicus* sp. nov., and *Macrococcus bovicus* sp. nov. and *Macrococcus carouselicus* sp. nov. *Int J Syst Bacteriol*, 48 Pt 3:859–877, Jul 1998.

G. Kozianowski, F. Canganella, F. Rainey, H. Hippe, and G. Antranikian. Purification and characterization of thermostable pectate-lyases from a newly isolated thermophilic bacterium, *Thermoanaerobacter italicus* sp. nov. *Extremophiles*, 1:171–182, Nov 1997.

S. Krishnamurthi, T. Chakrabarti, and E. Stackebrandt. Re-examination of the taxonomic position of *Bacillus silvestris* Rheims et al. 1999 and proposal to transfer it to *Solibacillus* gen. nov. as *Solibacillus silvestris* comb. nov. *Int J Syst Evol Microbiol*, 59:1054–1058, May 2009.

C. Lai, B. Males, P. Dougherty, P. Berthold, and M. Listgarten. *Centipeda periodontii* gen. nov., sp. nov. from human periodontal lesions. *Int J Syst Evol Microbiol*, 33(3):628–635, Jul 1983.

Y. Lee, M. Jain, C. Lee, S. Lowe, and J. Zeikus. Taxonomic distinction of saccharolytic thermophilic anaerobes: description of *Thermoanaerobacterium xyanolyticum* gen. nov., sp. nov., and *Thermoanaerobacterium saccharolyticum* gen. nov., sp. nov.; reclassification of *Thermoanaerobium brockii*, *Clostridium thermosulfurogenes*, and *Clostridium thermohydrosulfuricum* e100-69 as *Thermoanaerobacter brockii* comb. nov., *Thermoanaerobacterium thermo-*

sulfurigenes comb. nov., respectively; and transfer of *Clostridium thermohydrosulfuricum* 39e. 43(1):41–51, 1993.

M. Lindström and H. Korkeala. Laboratory diagnostics of botulism. *Clin Microbiol Rev*, 19: 298–314, Apr 2006.

C. Liu, S. Finegold, Y. Song, and P. Lawson. Reclassification of *Clostridium coccoides*, *Ruminococcus hansenii*, *Ruminococcus hydrogenotrophicus*, *Ruminococcus luti*, *Ruminococcus productus* and *Ruminococcus schinkii* as *Blautia coccoides* gen. nov., comb. nov., *Blautia hansenii* comb. nov., *Blautia hydrogenotrophica* comb. nov., *Blautia luti* comb. nov., *Blautia producta* comb. nov., *Blautia schinkii* comb. nov. and description of *Blautia wexlerae* sp. nov., isolated from human faeces. *Int J Syst Evol Microbiol*, 58:1896–1902, Aug 2008.

J. Lu, Y. Nogi, and H. Takami. *Oceanobacillus iheyensis* gen. nov., sp. nov., a deep-sea extremely halotolerant and alkaliphilic species isolated from a depth of 1050 m on the Iheya ridge. *FEMS Microbiol Lett*, 205:291–297, Dec 2001.

M. Marounek, K. Fliegrova, and S. Bartos. Metabolism and some characteristics of ruminal strains of *Megasphaera elsdenii*. *Appl Environ Microbiol*, 55:1570–1573, Jun 1989.

D. Miller, G. Suen, D. Bruce, A. Copeland, J. Cheng, C. Detter, L. Goodwin, C. Han, L. Hauser, M. Land, A. Lapidus, S. Lucas, L. Meincke, S. Pitluck, R. Tapia, H. Teshima, T. Woyke, B. Fox, E. Angert, and C. Currie. Complete genome sequence of the cellulose-degrading bacterium *Cellulosilyticum lentocellum*. *J Bacteriol*, 193:2357–2358, May 2011.

C. Moon, D. Pacheco, W. Kelly, S. Leahy, D. Li, J. Kopecny, and G. Attwood. Reclassification of *Clostridium proteoclasticum* as *Butyrivibrio proteoclasticus* comb. nov., a butyrate-producing ruminal bacterium. *Int J Syst Evol Microbiol*, 58:2041–2045, Sep 2008.

W. Moore, J. Johnson, and L. Holdeman. Emendation of bacteroidaceae and *Butyrivibrio* and descriptions of *Desulfomonas* gen. nov. and ten new species in the genera *Desulfomonas*, *Butyrivibrio*, *Eubacterium*, *Clostridium*, and *Ruminococcus*. *Int. J. Syst. Bacteriol.*, 26:238–252, 1976.

D. Murdoch and H. Shah. Reclassification of *Peptostreptococcus magnus* (prevot 1933) holdeman and moore 1972 as *Finegoldia magna* comb. nov. and *Peptostreptococcus micros* (prevot 1933) smith 1957 as *Micromonas micros* comb. nov. *Anaerobe*, 5(5):555–559, Oct 1999.

L. Nakamura, M. Roberts, and F. Cohan. Relationship of *Bacillus subtilis* clades associated with strains 168 and W23: a proposal for *Bacillus subtilis* subsp. *subtilis* subsp. nov. and *Bacillus subtilis* subsp. *spizizenii* subsp. nov. *Int J Syst Bacteriol*, 49 Pt 3:1211–1215, Jul 1999.

S. Naser, F. Thompson, B. Hoste, D. Gevers, K. Vandemeulebroecke, I. Cleenwerck, C. Thompson, M. Vancanneyt, and J. Swings. Phylogeny and identification of *Enterococci* by atpA gene sequence analysis. *J Clin Microbiol*, 43:2224–2230, May 2005.

T. Nazina, T. Tourova, A. Poltaraus, E. Novikova, A. Grigoryan, A. Ivanova, A. Lysenko, V. Petrunyaka, G. Osipov, S. Belyaev, and M. Ivanov. Taxonomic study of aerobic thermophilic bacilli: descriptions of *Geobacillus subterraneus* gen. nov., sp. nov. and *Geobacillus uzenensis* sp. nov. from petroleum reservoirs and transfer of *Bacillus stearothermophilus*, *Bacillus thermocatenulatus*, *Bacillus thermoleovorans*, *Bacillus kaustophilus*, *Bacillus thermodenitrificans* to *Geobacillus* as the new combinations *G. stearothermophilus*, *G. th. Int J Syst Evol Microbiol*, 51:433–446, Mar 2001.

I. Ntaikou, H. Gavala, M. Kornaros, and G. Lyberatos. Hydrogen production from sugars and sweet sorghum biomass using *Ruminococcus albus*. *Int J Hydrogen Energ*, 33:1153–1163, 2008.

S. O-Thong, P. Prasertsan, D. Karakashev, and I. Angelidaki. Thermophilic fermentative hydrogen production by the newly isolated *Thermoanaerobacterium thermosaccharolyticum* psu-2. *Int J Hydrogen Energy*, 33:1204–1214, 2008.

R. Onyenwoke, V. Kevbrin, A. Lysenko, and J. Wiegel. *Thermoanaerobacter pseudethanolicus* sp. nov., a thermophilic heterotrophic anaerobe from Yellowstone National Park. *Int J Syst Evol Microbiol*, 57:2191–2193, Oct 2007.

A. Palop, I. Alvarez, J. Raso, and S. Condón. Heat resistance of *Alicyclobacillus acidocaldarius* in water, various buffers, and orange juice. *J Food Prot*, 63:1377–1380, Oct 2000.

D. Paredes-Sabja, B. Setlow, P. Setlow, and M. Sarker. Characterization of *Clostridium perfringens* spores that lack spoVA proteins and dipicolinic acid. *J Bacteriol*, 190:4648–4659, Jul 2008.

D. Paredes-Sabja, A. Shen, and J. Sorg. *Clostridium difficile* spore biology: sporulation, germination, and spore structural proteins. *Trends Microbiol*, 22:406–416, Jul 2014.

S. Park, S. Park, and S. Choi. Characterization of sporulation histidine kinases of *Paenibacillus polymyxa*. *Res Microbiol*, 163:272–278, May 2012.

G. Patel, A. Khan, B. Agnew, and J. Colvin. Isolation and characterization of an anaerobic, cellulolytic microorganism, *Acetivibrio cellulolticus* gen. nov., sp. nov. *Int J Syst Evol Microbiol*, 30 (1):179–185, Jan 1980.

E. Petitdemange, F. Caillet, J. Giallo, and C. Gaudin. *Clostridium cellulolyticum* sp. nov., a cellulolytic, mesophilic species from decayed grass. *Int. J. Syst. Bacteriol.*, 34:155–159, 1984.

E. Pikuta, A. Lysenko, N. Chuvilskaya, U. Mendrock, H. Hippe, N. Suzina, D. Nikitin, G. Osipov, and K. Laurinavichius. *Anoxybacillus pushchinensis* gen. nov., sp. nov., a novel anaerobic, alkaliphilic, moderately thermophilic bacterium from manure, and description of *Anoxybacillus flavitherms* comb. nov. *Int J Syst Evol Microbiol*, 50 Pt 6:2109–2117, Nov 2000.

G. Reddy, J. Prakash, M. Vairamani, S. Prabhakar, G. Matsumoto, and S. Shivaji. *Planococcus antarcticus* and *Planococcus psychrophilus* spp. nov. isolated from cyanobacterial mat samples collected from ponds in Antarctica. *Extremophiles*, 6:253–261, Jun 2002.

W. Robertson, P. Franzmann, and B. Mee. Spore-forming, *Desulfosporosinus*-like sulphate-reducing bacteria from a shallow aquifer contaminated with gasoline. *J Appl Microbiol*, 88: 248–259, Feb 2000.

M. Salinas, M. Fardeau, P. Thomas, J. Cayol, B. Patel, and B. Ollivier. *Mahella australiensis* gen. nov., sp. nov., a moderately thermophilic anaerobic bacterium isolated from an Australian oil well. *Int J Syst Evol Microbiol*, 54:2169–2173, Nov 2004.

Y. Sekiguchi, Y. Kamagata, K. Nakamura, A. Ohashi, and H. Harada. *Syntrophothermus lipocalidus* gen. nov., sp. nov., a novel thermophilic, syntrophic, fatty-acid-oxidizing anaerobe which utilizes isobutyrate. *Int J Syst Evol Microbiol*, 50 Pt 2:771–779, Mar 2000.

J. Seo, H. Kim, G. Jung, M. Nam, J. Chung, J. Kim, J. Yoo, C. Kim, and O. Kwon. Psychrophilicity of *Bacillus psychrosaccharolyticus*: a proteomic study. *Proteomics*, 4:3654–3659, Nov 2004.

E. Shelobolina, K. Nevin, J. Blakeney-Hayward, C. Johnsen, T. Plaia, P. Krader, T. Woodard, D. Holmes, C. Vanpraagh, and D. Lovley. *Geobacter pickeringii* sp. nov., *Geobacter argillaceus* sp. nov. and *Pelosinus fermentans* gen. nov., sp. nov., isolated from subsurface kaolin lenses. *Int J Syst Evol Microbiol*, 57:126–135, Jan 2007.

O. Shida, H. Takagi, K. Kadowaki, and K. Komagata. Proposal for two new genera, *Brevibacillus* gen. nov. and *Aneurinibacillus* gen. nov. *Int J Syst Bacteriol*, 46:939–946, Oct 1996.

J. Sieber, D. Sims, C. Han, E. Kim, A. Lykidis, A. Lapidus, E. McDonnald, L. Rohlin, D. Culley, R. Gunsalus, and M. McInerney. The genome of *Syntrophomonas wolfei*: new insights into syntrophic metabolism and biohydrogen production. *Environ Microbiol*, 12:2289–2301, Aug 2010.

T. Sokolova, J. González, N. Kostrikina, N. Chernyh, T. Slepova, E. Bonch-Osmolovskaya, and F. Robb. *Thermosinus carboxydivorans* gen. nov., sp. nov., a new anaerobic, thermophilic, carbon-monoxide-oxidizing, hydrogenogenic bacterium from a hot pool of yellowstone national park. *Int J Syst Evol Microbiol*, 54:2353–2359, Nov 2004.

F. Soriano, R. Fernández-Roblas, R. Calvo, and G. García-Calvo. *In vitro* susceptibilities of aerobic and facultative non-spore-forming gram-positive bacilli to HMR 3647 (RU 66647) and 14 other antimicrobials. *Antimicrob Agents Chemother*, 42:1028–1033, May 1998.

S. Spring, M. Visser, M. Lu, A. Copeland, A. Lapidus, S. Lucas, J. Cheng, C. Han, R. Tapia, L. Goodwin, S. Pitluck, N. Ivanova, M. Land, L. Hauser, F. Larimer, M. Rohde, M. Göker, J. Detter, N. Kyrpides, T. Woyke, P. Schaap, C. Plugge, G. Muyzer, J. Kuever, I. Pereira, S. Parshina, R. Bernier-Latmani, A. Stams, and H. Klenk. Complete genome sequence of the sulfate-reducing firmicute *Desulfotomaculum ruminis* type strain (dl(t)). *Stand Genomic Sci*, 7: 304–319, Dec 2012.

E. Stackebrandt, H. Pohla, R. Kroppenstedt, H. Hippe, and C. R. Woese. 16S rRNA analysis of *Sporomusa*, *Selenomonas*, and *Megasphaera*: on the phylogenetic origin of gram-positive eubacteria. *Arch Microbiol*, 143:270–276, 1985.

T. Stadtman and L. McClung. *Clostridium sticklandii* nov. spec. *J Bacteriol*, 73:218–219, Feb 1957.

T. Stanton and D. C. Savage. *Roseburia cecicola* gen. nov., a motile obligately anaerobic bacterium from a mouse cecum. *Int J Syst Bacteriol*, 33:618–627, 1983.

E. Steiner, A. Dago, D. Young, J. Heap, N. Minton, J. Hoch, and M. Young. Multiple orphan histidine kinases interact directly with Spo0A to control the initiation of endospore formation in *Clostridium acetobutylicum*. *Mol Microbiol*, 80:641–654, May 2011.

Y. Tan, M. Ayob, M. Osman, and K. Matthews. Antibacterial activity of different degree of hydrolysis of palm kernel expeller peptides against spore-forming and non-spore-forming bacteria. *Lett Appl Microbiol*, 53:509–517, Nov 2011.

D. Taras, R. Simmering, M. Collins, P. Lawson, and M. Blaut. Reclassification of *Eubacterium formicigenerans* Holdeman and Moore 1974 as *Dorea formicigenerans* gen. nov., comb. nov., and description of *Dorea longicatena* sp. nov., isolated from human faeces. *Int J Syst Evol Microbiol*, 52:423–428, Mar 2002.

D. Tena, N. Martínez, J. Casanova, J. García, E. Román, M. Medina, and J. Sáez-Nieto. Possible *Exiguobacterium sibiricum* skin infection in human. *Emerg Infect Dis*, 20:2178–2179, Dec 2014.

C. Thompson, R. Vier, A. Mikaelyan, T. Wienemann, and A. Brune. '*Candidatus* arthromitus' revised: segmented filamentous bacteria in arthropod guts are members of *Lachnospiraceae*. *Environ Microbiol*, 14:1454–1465, Jun 2012.

E. Tocheva, E. Matson, D. Morris, F. Moussavi, J. Leadbetter, and G. Jensen. Peptidoglycan remodeling and conversion of an inner membrane into an outer membrane during sporulation. *Cell*, 146:799–812, Sep 2011.

I. Vishniakov, S. Levitski, and S. Borkhsenius. Effect of heat shock on cells of phytopathogenic mycoplasma *Acholeplasma laidlawii* pg-8a. *Tsitologiia*, 57:5–13, 2015.

P. Vos, G. Garrity, D. Jones, N.R. Krieg, W. Ludwig, and F.A. Rainey. *Bergey's Manual of Systematic Bacteriology*. Springer-Verlag New York, 2009. ISBN 9780387950419.

T. Warnick, B. Methé, and S. Leschine. *Clostridium phytofermentans* sp. nov., a cellulolytic mesophile from forest soil. *Int J Syst Evol Microbiol*, 52:1155–1160, Jul 2002.

Y. Watanabe, F. Nagai, and M. Morotomi. Characterization of *Phascolarctobacterium succinatutens* sp. nov., an asaccharolytic, succinate-utilizing bacterium isolated from human feces. *Appl Environ Microbiol*, 78:511–518, Jan 2012.

F. Widdel and N. Pfennig. A new anaerobic, sporing, acetate-oxidizing, sulfate-reducing bacterium, *Desulfotomaculum* (emend.) *acetoxidans*. *Arch Microbiol*, 112:119–122, Feb 1977.

K. Willquist, A. Zeidan, and E. van Niel. Physiological characteristics of the extreme thermophile *Caldicellulosiruptor saccharolyticus*: an efficient hydrogen cell factory. *Microb Cell Fact*, 9:89, Nov 2010.

M. Wu, Q. Ren, A. Durkin, S. Daugherty, L. Brinkac, R. Dodson, R. Madupu, S. Sullivan, J. Kolonay, D. Haft, W. Nelson, L. Tallon, K. Jones, L. Ulrich, J. Gonzalez, I. Zhulin, F. Robb,

and J. Eisen. Life in hot carbon monoxide: the complete genome sequence of *Carboxydothermus hydrogenoformans* z-2901. *PLoS Genet*, 1:e65, Nov 2005.

D. Xing, N. Ren, Q. Li, M. Lin, A. Wang, and L. Zhao. *Ethanoligenens harbinense* gen. nov., sp. nov., isolated from molasses wastewater. *Int J Syst Evol Microbiol*, 56:755–760, Apr 2006.

Q. Ye, Y. Roh, S. Carroll, B. Blair, J. Zhou, C. Zhang, and M. Fields. Alkaline anaerobic respiration: isolation and characterization of a novel alkaliphilic and metal-reducing bacterium. *Appl Environ Microbiol*, 70:5595–5602, Sep 2004.

N. Yutin and M. Y. Galperin. A genomic update on clostridial phylogeny: Gram-negative spore formers and other misplaced clostridia. *Environ Microbiol*, 15:2631–41, Oct 2013.
