# Supplementary material for: Flexibility and constraint: Evolutionary remodeling of the sporulation initiation pathway in Firmicutes
Source: PLoS Genet. 2018 Sep 13;14(9):e1007470. doi: 10.1371/journal.pgen.1007470 (PMC6136694; doi:10.1371/journal.pgen.1007470)
Supplement: S1 Text — (PDF) [file pgen.1007470.s017.pdf]

S1 Text Comparison of the distribution of Spo0 Components in alternative phylogenies

Our analysis of 83 representative genomes revealed genomes with a predicted Spo0 phosphorelay architecture throughout the Firmicutes phylum with a patchy phylogenetic distribution (Fig 3). To verify that our findings are not an artifact of the selection of genomes in our study set or the phylogenetic methods used, we took advantage of two recently published alternative Firmicutes phylogenies [Antunes et al., 2016; Yutin and Galperin, 2013], one of which is based on a much larger set of Firmicutes genomes [Antunes et al., 2016]. We scanned genomes in the Antunes et al. [2016] and Yutin and Galperin [2013] data sets for orthologs of Spo0 pathway components, using the same procedure described in the main text. The Spo0 architectures predicted in spore-forming genomes in both sets have patchy phylogenetic distributions (Figs S2 and S3), similar to those observed in our original analysis. In particular, in both data sets, we found phosphorelay architectures in both major taxonomic classes. The patchiness observed in all three trees is consistent with multiple changes in pathway architecture over the course of evolution.

All three trees were constructed from concatenated alignments of 45 to 50 ribosomal protein sequences, although the exact set of ribosomal proteins varies. The Yutin tree is based on 68 genomes selected to probe Clostridium species and to investigate the phylogenetic placement of the Negativicutes (Selenomonadales and Veillonellaceae). This tree includes a well-sampled set of Class Clostridia genomes, with particular focus on the Clostridium genomes spanning the Clusters defined by Collins et al. [1994] based on 16S rRNA, as well as two outgroup sequences used to root the tree. The Antunes tree is based on a 205 Firmicutes genomes selected to examine the evolutionary history of mono- and diderm cellular envelopes. This dataset covers the entire Firmicutes phylum extensively, including many species in two large clades of non-sporulating species lacking Spo0A (Lactobacilliales and Veillonellaceae) that were not well-represented in our data set (shown as collapsed clades in Fig S3). In addition, 13 outgroup species were included to root the tree (not shown in Fig S3).

At the highest taxonomic ranks, the Yutin and Antunes tree topologies are similar to each other and to our tree (Fig 3), despite differences in pre-processing, tree reconstruction methodology, and taxon sampling. There are minor differences in the inferred relationships lower in the taxonomy, especially with respect to branches with weak sequence support. The remainder of this supplementary text examines the positions of subgroups that differ across the three trees. In each case, we discuss the implications of those differences for our hypotheses regarding the ancestral pathway or patchy distribution.

S1.1 Halanaerobiales and Natranaerobiales

The Halanaerobiales and Natranaerobiales are anaerobic, halophilic extremophiles [Mesbah et al., 2007; Roush et al., 2014]. The Antunes data set includes all currently available, fully sequenced genomes from these taxa; i.e., the genomes of *Natranaerobius thermophilus* and five members of the Halanaerobiales. The Yutin data set includes *N. thermophilus* and one Halanaerobiales genome. No genomes from these taxa were included in our data set.

Analysis of these genomes for Spo0 components revealed putative Spo0B and Spo0F orthologs in the only available Natranaerobiales genome (*Natranaerobius thermophilus*), which is consistent with a phosphorelay, although this species is a reported non-spore former [Mesbah et al., 2007]. One spore-forming member of the Natranaerobiales order has been reported, *Natranaerobaculum magdiense* [Zavarzina et al., 2013], but a whole genome sequence for this species has not been published.

All Halanaerobiales analyzed encode Spo0A and at least one PAS-containing histidine kinase, but neither Spo0F, nor Spo0B. Again, all of the Halanaerobiales species included in either the Antunes or Yutin tree are considered to be asporogenous [Mavromatis et al., 2009; Oren et al., 1991; Sikorski et al., 2010; Vos et al., 2009; Zhilina et al., 1996]. However, several species within the order Halanaerobiales have been reported to form spores, including *Halonatronum saccharophilum* [Zhilina et al., 2012], *Fuchsiella ferrireducens* [Zhilina et al., 2015], and *Natroniella acetigena* [Zhilina et al., 1996]. The genus *Sporohalobacter* was initially reported to form spores [Oren et al., 1991], but subsequent characterization called this result into question as no growth was obtained following heat treatment [Ben Abdallah et al., 2015]. Swollen end cells were characterized as pre-spore-like structures, but no spores were observed via microscopy. These experimental differences could be due to the conditions tested or different assessment of what constitutes a spore. Of spore-forming Halanaerobiales, the only available genome is *Halonatronum saccharophilum*, which does not encode Spo0F or Spo0B orthologs according to our genome neighborhood conservation method.

Genomes from Halanaerobiales and Natranaerobiales form a clade in both recently published trees, but the location of that clade, in relation to other Firmicutes differs. In the Antunes tree, these taxa are basal to the divergence of Classes Bacilli and Clostridia. Since this placement makes them the earliest branching clade within the Firmicutes, the presence of an apparent phosphorelay architecture in *N. thermophilus* supports the hypothesis that the emergence of the Spo0 phosphorelay predates the divergence of the Clostridia and Bacilli.

In the Yutin tree, the clade (which includes one representative of each order, *Natranaerobius thermophilus* and *Halothermothrix oreni*) is one of several descendants of a polytomy at the base

of Class Clostridia. Since this node is unresolved, this placement neither supports, nor refutes our prediction that the common ancestor of the Clostridia and Bacilli likely encoded a phosphorelay Spo0 pathway.

Regardless of their phylogenetic placement in the context of other species, the presence of a phosphorelay in the Natranaerobiales and a direct phosphorylation architecture in the Halanaerobiales adds to the patchy distribution of Spo0 architectures observed throughout the Phylum, requiring an additional remodeling event to explain the present-day phylogenetic distribution. The placement of these sister taxa in the two recently published trees does not contradict the hypothesis that the phosphorelay architecture was present in the common ancestor of the Bacilli or Clostridia; moreover, the evidence from one of those studies suggests that it predates that common ancestor.

S1.2 *Alkaliphilus*, *Gottschalkia*, and Sporulating Peptostreptococcaceae

All three trees predict a clade consisting of species from the families Clostridiaceae (only genus *Alkaliphilus*), Eubacteriaceae (genera *Acetobacterium*, *Eubacterium*), Peptoniphilaceae (genera *Finegoldia*, *Anaerococcus*), Incertae sedis XI (*Gottschalkia*), and Peptostreptococcaceae (genera *Clostridiodes*, *Peptoclostridium*, *Filifactor*). In all three trees, this clade is a sister taxon to the *Clostridium sensu stricto*. Many genera within this clade are reportedly asporogenous [Galperin et al., 2012]. The key exceptions are four species in the genera *Alkaliphilus* [Cao et al., 2003], *Gottschalkia* [Yutin and Galperin, 2013], and *Clostridioides* [Lawson et al., 2016]. Each tree contains a different subset of these four spore-formers: *Clostridioides difficile* is represented in all trees, *Alkaliphilus metalliredigens* in the Antunes tree and our tree, *Gottschalkia acidurici* in the Yutin tree and our tree, and *Alkaliphilus oremlandii* only in the Antunes tree.

All trees agree on the relationships between these taxa: *Alkaliphilus* is a sister taxon to *Clostridiodes difficile* and other Peptostreptococcaceae, when *Gottschalkia* is not present [Antunes et al., 2016], and vice versa [Yutin and Galperin, 2013]. When both are present (as in our tree), *Gottschalkia* is basal to a clade that includes both *Alkaliphilus* and *Clostridiodes*. Notably, there are asporogenous species interleaved between these taxa in all three trees.

These species are particularly interesting because, although closely related, they have different predicted Spo0 architectures. *A. metalliredigens* and *G. acidurici* were found to encode homologs of Spo0F and Spo0B and are therefore likely to initiate sporulation via a phosphorelay. No phosphorelay homologs were observed in either *A. oremlandii* or *C. difficile*, suggesting that these sporulating species have a direct phosphorylation Spo0 pathway. The presence of the phosphorelay homologs in *G. acidurici* and *A. metalliredigens* suggests that the phosphorelay has persisted despite repeated losses of Spo0F and Spo0B and/or sporulation within closely related taxa. This

mixed distribution implies multiple transitions from phosphorelay to direct phosphorylation architecture within the Clostridiales, one at the base of each divergent group. This inference is supported by all three trees.

S1.3 Predicted architectures within Class Bacilli

Each tree has a different set of species from Class Bacilli, but the results of these differences are not at variance with the observations made here. Homologs of Spo0F and Spo0B were detected by our methods in all genomes in spore-forming Bacilli represented in the three data sets, with the exception of two *Erysipelaclostridium* genomes, two *Paenibacillus* genomes and the genome of *Sporolactobacillus inulinus*. Each of these exceptions is treated below.

Erysipelatoclostridium: The genomes of *Erysipelatoclostridium ramosum DSM 1402* and *Erysipelatoclostridium spiroforme DSM 1552* both encode Spo0A; *E. spiroforme* additionally encodes an orphan kinase. Spore formation in these species has been described as “rare or absent” [Kaneuchi et al., 1979; Lavigne et al., 2003; Yutin and Galperin, 2013]. No Erysipelatrichiaceae species were included in the Antunes data set. In the Yutin tree, these genomes are basal to all other Bacilli. In our tree, these genomes are located within the Bacillaceae clade. However, since the Yutin data set does not include any early branching genomes in class Bacilli (e.g. Paenibacillaceae), the branching order in the two trees is consistent.

***Paenibacillus*:** Both Paenibacillaceae genomes, *Paenibacillus polymyxa* and *Brevibacillus brevis*, included in our representative set possess all phosphorelay components. In our tree, these genomes form a clade that is a sister taxon to the Bacillaceae. These taxa are not represented in the Yutin tree. The Antunes tree includes the genomes of six members of the Paenibacillaceae, including genomes from the genera *Desmospora*, *Brevibacillus*, *Paenibacillus*, and *Thermobacillus*. These species are phylogenetically placed basal to the Bacillaceae, though paraphyletically.

Spo0B was not identified in two of these species, *Paenibacillus mucilaginosa* and *Paenibacillus sp.* Y412MC10. In both cases, inspection of the genome neighborhood of these two species reveals a hypothetical protein with the similar in sequence and domain content to Spo0B, though it appears to be missing a stop codon. This could be a loss of function mutation or due to an error in sequencing or assembly. This hypothetical protein was accepted as sufficient evidence for the presence of Spo0B in our analysis.

If Spo0B is truly a pseudogene in these species, this could indicate either the loss of sporulation or gain of the ability to sporulate via direct phosphorylation of Spo0A in these individuals. Interestingly, the *Paenibacillus polymyxa* kinase PP_1077 has been reported to directly phosphorylate *P. polymyxa* Spo0A when heterologously expressed in a *B. subtilis* mutant lacking Spo0B but not

lacking any of the *B. subtilis* sporulation kinases [Park et al., 2012]. Spo0 architectures in these species warrant further investigation.

***Sporolactobacillus*:** *S. inulinus* is present in the Antunes data set, but was not included in either our tree or the Yutin tree. The Antunes tree places this species basal to the Bacillaceae, diverging after the Paenibacillaceae. Spo0F was not identified by conserved genome neighborhood in *S. inulinus*, although *S. inulinus* is reported to produce endospores [Kitahara and Lai, 1967]. However, inspection of predicted RRs lacking an output domain in that species did reveal a possible candidate (SINU_10335). This protein aligns well to known and predicted Spo0F sequences and has specificity residues typical of Spo0F (QGILEVD), although it is not encoded in the proximity of any of the Spo0F neighborhood markers. All other single-domain RRs in this species had less sequence similarity to Spo0Fs and specificity residues that did not reflect the Spo0F signature (Fig 5). This was accepted as sufficient evidence for the presence of Spo0F in our analysis.

S1.4 Ruminococcaceae and Lachnospiraceae

Species from the Ruminococcaceae and Lachnospiraceae are well-sampled in all three trees, however the relationship of these two families with respect to the Clostridiaceae varies slightly. In our tree and the Antunes tree, they are sister taxa to the Clostridiaceae, while the relationship between these three clades is not resolved in the Yutin tree. All sporogenous members of the Ruminococcaceae and Lachnospiraceae families are predicted to encode a direct phosphorylation architecture. If these two clades represent distinct lineages that are not sister taxa, as possible in the Yutin tree, then an additional transition between phosphorelay and direct phosphorylation architecture is required to explain the phylogenetic distribution of Spo0 pathway architectures in these species.

S1.5 Spore-formers with symbiotic or syntrophic life styles

Several spore-forming species in the Antunes tree have symbiotic or syntrophic lifestyles, including members of the genera *Symbiobacterium* [Ohno et al., 2000], *Thermaerobacter* [Han et al., 2010], *Tepidanaerobacter* [Westerholm et al., 2011], and *Thermosediminibacter* [Pitluck et al., 2010]. Only *Symbiobacterium thermophilus* was included in the Yutin tree and none were included in our tree.

Candidate Spo0F and Spo0B orthologs were not found in the *Symbiobacterium* and *Thermaerobacter* genomes, but were found in the closely related species, *Sulfobacillus acidophilus DSM 10332*, which has a typical free-living lifestyle [Norris et al., 1996]. The sister taxa *Tepidanaerobacter* and *Thermosediminibacter* encode orthologs of Spo0B, but not Spo0F, and lack orphan kinases. Of these species, all but *Thermosediminibacter oceani* have been observed to produce

spores.

Interpreting the Spo0 pathway architectures in these species is complicated by their symbiotic nature. For example, *Symbiobacterium thermophilum* displays marked growth dependence on microbial commensalism with *Bacillus sp.* strain S [Ueda et al., 2004]. Similarly, sporulation increases from 0.1% to 20% when cultured in a dialysis flask with a constant influx of media used by *Bacillus sp.* strain S [Ueda et al., 2004], suggesting an apparent reliance on external factors to initiate sporulation. These external factors could be small signaling molecules or, potentially, proteins encoded by *Bacillus sp.* strain S that facilitate the phosphorylation of Spo0A in *S. thermophilum*. Further work on the factors on which these syntrophic and symbiotic bacteria rely may reveal the mechanism of initiation of sporulation in these species. Taking a conservative stance, we do not interpret the absence of genes encoding Spo0 pathway proteins to be evidence of an alternative Spo0 pathway architecture in symbiotic or syntrophic strains.

Bibliography

L. C. Antunes, D. Poppleton, A. Klingl, A. Criscuolo, B. Dupuy, C. Brochier-Armanet, C. Beloin, and S. Gribaldo. Phylogenomic analysis supports the ancestral presence of LPS-outer membranes in the Firmicutes. *Elife*, 5, Aug 31 2016.

M. Ben Abdallah, F. Karray, N. Mhiri, J. L. Cayol, J. L. Tholozan, D. Alazard, and S. Savadi. Characterization of *Sporohalobacter salinus* sp. nov., an anaerobic, halophilic, fermentative bacterium isolated from a hypersaline lake. *Int J Syst Evol Microbiol*, 65(Pt 2):543–8, Feb 2015.

X. Cao, X. Liu, and X. Dong. *Alkaliphilus crotonatoxidans* sp. nov., a strictly anaerobic, crotonate-dismutating bacterium isolated from a methanogenic environment. *Int J Syst Evol Microbiol*, 53 (Pt 4):971–5, Jul 2003.

M. D. Collins, P. A. Lawson, A. Willems, J. J. Cordoba, J. Fernandez-Garayzabal, P. Garcia, Cai J., H. Hippe, and Farrow J. A. The phylogeny of the genus *Clostridium*: proposal of five new genera and eleven new species combinations. *Int J Syst Bacteriol*, 44(4):812–26, Oct 1994.

M. Galperin, S. Mekhedov, P. Puigbo, S. Smirnov, Y. Wolf, and D. Rigden. Genomic determinants of sporulation in Bacilli and Clostridia: towards the minimal set of sporulation-specific genes. *Environ Microbiol*, Jul 2012.

C. Han, W. Gu, X. Zhang, A. Lapidus, M. Nolan, A. Copeland, S. Lucas, T. G. Del Rio, H. Tice, JF. Cheng, R. Tapia, L. Goodwin, S. Pitluck, I. Pagani, N. Ivanova, K. Mavromatis, N. Mikhailova, A. Pati, A. Chen, K. Palaniappan, M. Land, L. Hauser, YJ. Chang, C. D. Jeffries, S. Schneider, M. Rohde, M. Goker, R. Pukall, T. Woyke, J. Bristow, JA. Eisen, V. Markowitz, P. Hugenholtz, N. C. Kyrpides, HP. Klenk, and J. C. Detter. Complete genome sequence of *Thermaerobacter marianensis* type strain (7p75a). *Stand Genomic Sci*, 3(3):337–45, Dec 15 2010.

C. Kaneuchi, T. Miyazato, T. Shinjo, and T. Mitsuoka. Taxonomic study of helically coiled, spore-forming anaerobes isolated from the intestines of humans and other animals: *Clostridium cocleatum* sp. nov. and *Clostridium spiroforme* sp. nov. *Int J Syst Evol Microbiol*, 29(1):1–12, 1979.

K. Kitahara and C. L. Lai. On the spore formation of *Sporolactobacillus inulinus*. *J Gen Appl Microbiol*, 197-203, 13 1967.

J. P. Lavigne, N. Bouziges, A. Sotto, J. L. Leroux, and S. Michaux-Charachon. Spondylodiscitis due to *Clostridium ramosum* infection in an immunocompetent elderly patient. *J Clin Microbiol*, 41(5):2223–6, May 2003.

P. Lawson, D. Citron, K. Tyrrell, and S. Finegold. Reclassification of *Clostridium difficile* as *Clostridioides difficile* (Hall and O'Toole 1935) Prévot 1938. *Anaerobe*, 40:95–99, Aug 2016.

K. Mavromatis, N. Ivanova, I. Anderson, A. Lykidis, S.D. Hooper, H. Sun, V. Kunin, A. Lapidus, P. Hugenholtz, B. Patel, and N. C. Kyrpides. Genome analysis of the anaerobic thermohalophilic bacterium *Halothermothrix orenii*. *PLoS One*, e4192, 4(1) 2009.

N. Mesbah, D. Hedrick, A. Peacock, M. Rohde, and J. Wiegel. *Natranaerobius thermophilus* gen. nov., sp. nov., a halophilic, alkalithermophilic bacterium from soda lakes of the Wadi An Natrun, Egypt, and proposal of Natranaerobiaceae fam. nov. and Natranaerobiales ord. nov. *Int J Syst Evol Microbiol*, 57(11):2507–2512, Nov 2007.

P. R. Norris, D. A. Clark, J. P. Owen, and S. Waterhouse. Characteristics of *Sulfobacillus acidophilus* sp. nov. and other moderately thermophilic mineral-sulphide-oxidizing bacteria. *Microbiology*, 142(Pt 4):775–83, Apr 1996.

M. Ohno, H. Shiratori, M. J. Park, Y. Saitoh, Y. Kumon, N. Yamashita, A. Hirata, H. Nishida, K. Ueda, and T. Beppu. *Symbiobacterium thermophilum* gen. nov., sp. nov., a symbiotic thermophile that depends on co-culture with a *Bacillus* strain for growth. *Int J Syst Evol Microbiol*, 50(Pt 5):1829–32, Sep 2000.

A. Oren, P. Gurevich, and Y. Henis. Reduction of nitrosubstituted aromatic compounds by the halophilic anaerobic eubacteria *Haloanaerobium praevalens* and *Sporohalobacter marismortui*. *Appl Environ Microbiol*, 57(11):3367–70, Nov 1991.

S. Park, S. Park, and S. Choi. Characterization of sporulation histidine kinases of *Paenibacillus polymyxa*. *Res Microbiol*, 163:272–278, May 2012.

S. Pitluck, M. Yasawong, C. Munk, M. Nolan, A. Lapidus, S. Lucas, T. Glavina Del Rio, H. Tice, J. F. Cheng, D. Bruce, C. Detter, R. Tapia, C. Han, L. Goodwin, K. Liolios, N. Ivanova, K. Mavromatis, N. Mikhailova, A. Pati, A. Chen, K. Palaniappan, M. Land, L. Hauser, Y. J. Chang, C. D. Jeffries, M. Rohde, S. Spring, J. Sikorski, M. Goker, T. Woyke, J. Bristow, J. A. Eisen, V. Markowitz, P. Hugenholtz, N. C. Kyrpides, and Klenk H. P. Complete genome sequence of *Thermosediminibacter oceani* type strain (JW/IW-1228P). *Stand Genomic Sci*, 3(2): 108–16, Sep 28 2010.

D. Roush, D. Elias, and M. Mormile. Metabolic capabilities of the members of the order Halanaerobiales and their potential biotechnological applications. *Curr Biotech*, 3(1):3–9, 2014.

J. Sikorski, A. Lapidus, O. Chertkov, S. Lucas, A. Copeland, T. Glavina Del Rio, M. Nolan, H. Tice, J. F. Cheng, C. Han, E. Brambilla, S. Pitluck, K. Liolios, N. Ivanova, K. Mavromatis, N. Mikhailova, A. Pati, D. Bruce, C. Detter, R. Tapia, L. Goodwin, A. Chen, K. Palaniappan, M. Land, L. Hauser, Y. J. Chang, C. D. Jeffries, M. Rohde, M. Goker, S. Spring, T. Woyke, J. Bristow, J. A. Eisen, V. Markowitz, P. Hugenholtz, N. C. Kyrpides, and Klenk H. P. Complete genome sequence of *Acetohalobium arabaticum* type strain (z-7288). *Stand Genomic Sci*, 3(1): 57–65, Aug 20 2010.

K. Ueda, A. Yamashita, J. Ishikawa, M. Shimada, T. O. Watsuji, K. Morimura, H. Ikeda, M. Hattori, and T. Beppu. Genome sequence of *Symbiobacterium thermophilum*, an uncultivable bacterium that depends on microbial commensalism. *Nucleic Acids Res*, 32(16):4937–44, Sep 21 2004.

P. Vos, G. Garrity, D. Jones, N.R. Krieg, W. Ludwig, and F.A. Rainey. *Bergey's Manual of Systematic Bacteriology*. Springer-Verlag New York, 2009. ISBN 9780387950419.

M. Westerholm, S. Roos, and A. Schnürer. *Tepidanaerobacter acetatoxydans* sp. nov., an anaerobic, syntrophic acetate-oxidizing bacterium isolated from two ammonium-enriched mesophilic methanogenic processes. *Syst Appl Microbiol*, 34(4):260–266, Jun 2011.

N. Yutin and M. Y. Galperin. A genomic update on clostridial phylogeny: Gram-negative spore formers and other misplaced clostridia. *Environ Microbiol*, 15:2631–41, Oct 2013.

D. G. Zavarzina, T. N. Zhilina, B. B. Kuznetsov, T. V. Kolganova, G. A. Osipov, M. S. Kotelev, and G. A. Zavarzin. *Natranaerobaculum magadiense* gen. nov., sp. nov., an anaerobic, alkalithermophilic bacterium from soda lake sediment. *Int J Syst Evol Microbiol*, 63(Pt 12):4456–61, Dec 2013.

T. N. Zhilina, G. A. Zavarzin, E. N. Detkova, and F. A. Rainey. *Natroniella acetigena* gen. nov., sp. nov., an extremely haloalkaliphilic, homoacetic bacterium: A new member of Haloanaerobiales. *Curr Microbiol*, 32(6):320–6, Jun 1996.

T. N. Zhilina, D. G. Zavarzina, A. N. Panteleeva, G. A. Osipov, N. A. Kostrikina, T. P. Tourova, and G. A. Zavarzin. *Fuchsiella alkaliacetigena* gen. nov., sp. nov., an alkaliphilic, lithoautotrophic homoacetogen from a soda lake. *Int J Syst Evol Microbiol*, 62(Pt 7):1666–73, Jul 2012.

T. N. Zhilina, D. G. Zavarzina, E. N. Detkova, E. O. Patutina, and B. B. Kuznetsov. *Fuchsiella ferrireducens* sp. nov., a novel haloalkaliphilic, lithoautotrophic homoacetogen capable of iron reduction, and emendation of the description of the genus *Fuchsiella*. *Int J Syst Evol Microbiol*, 65(8):2432–40, Aug 2015.
