# Supplementary material for: Flexibility and constraint: Evolutionary remodeling of the sporulation initiation pathway in Firmicutes
Source: PLoS Genet. 2018 Sep 13;14(9):e1007470. doi: 10.1371/journal.pgen.1007470 (PMC6136694; doi:10.1371/journal.pgen.1007470)
Supplement: S2 Text — (PDF) [file pgen.1007470.s018.pdf]

S2 Text Specificity Residue Similarity

In order to assess how the genetic determinants of specificity changed in the course of Spo0 pathway evolution, we compared the specificity residues in Spo0 pathway proteins between pathway architectures (phosphorelay versus direct phosphorylation). Within the set of phosphorelay proteins, we compared specificity residues between taxonomic groups (Bacilli versus Clostridia). In the main text, we presented a qualitative comparison (Fig 5). Here, we provide quantitative measures of the differences in specificity residues within and across architectures.

We quantify the differences in specificity residues for two sets of orthologous proteins as follows: The specificity residues of a set of n orthologous proteins can be represented as an n by l matrix, where l is the number of specificity sites (i.e., $l = 6$ for kinases and $l = 7$ for response receivers). Given two such matrices, the differences in specificity residues can be assessed with a site-by-site comparison. Given specificity residues from two sets of orthologs, we calculate a score

$$S_{1,2}(j) = \sum_a |f_1(a,j) - f_2(a,j)|$$

for site j, where the summation is over the 20 amino acids and $f_m(a,j)$ is defined to be the frequency of amino acid a at site j in set $m \in \{1,2\}$, if the frequencies of a in the two sets are significantly different at the α level, and $f_m(a,j) = 0$, otherwise. We consider the frequencies of a in Set 1 and Set 2 to be significantly different, if we can reject the null hypothesis that they represent the same underlying distribution. An overall score, $S_{1,2}$, for the difference of the amino acid frequency distributions in two sets is obtained by averaging $S_{1,2}(j)$ over all l sites.

We used the program TwoSampleLogo [Vacic et al., 2006] to obtain the values of $f_m(a,j)$ required to calculate $S_{1,2}(\alpha)$. This program includes a statistical framework for assessing whether residue frequencies are significantly different and offers a selection of statistical tests. We used the t-test. Given two sets of amino acids and a significance threshold, α, as input, `TwoSampleLogo -F TXT` reports for each site, the frequencies for each residue that has significantly different frequencies at the α level at that site. TwoSampleLogo also provides a visual comparison of two sets of specificity residues, again filtered by $p \leq \alpha$ at each site.

Using this technique, we calculated S_{BC}, the difference in the specificity residue distributions in Bacillar and Clostridial phosphorelay Spo0 proteins, using filtering with a p-value of $\alpha = 0.01$. To obtain insight into the remodeling events that resulted in the present-day distribution of Spo0 architectures, we also compared the specificity logos of orphan kinases and Spo0A from phosphorelays and direct phosphorylation architectures. We calculated the differences in the the specificity

residue distributions for Bacillar phosphorelays and all direct phosphorylation architectures (S_{BD}) and for Clostridial phosphorelays and all direct phosphorylation architectures (S_{CD}), separately. The quantitative assessment of these differences is summarized in S2Text.Table 1. The distributions of amino acids with significantly different frequencies at each site are presented visually in S2Text.Fig 1).

S2Text.Table. 1: Quantitative Comparison of Spo0 Specificity Logos ($p < 0.01$)

Spo0 component	Clostridial phosphorelay	Clostridial phosphorelay	Bacillar phosphorelay
	Bacillar phosphorelay	Direct Phosphorylation Architecture	
Orphan kinases	0.18	0.49	0.54
Spo0F	0.28		
Spo0B	0.44		
Spo0A	0.29	0.0	0.43

Spo0 Component	Bacillar phosphorelay	Clostridial phosphorelay	Bacillar phosphorelay
	Clostridial phosphorelay	Direct phosphorylation Architecture	
Orphan Kinases	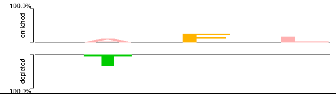	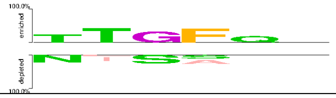	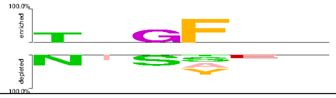
Spo0F	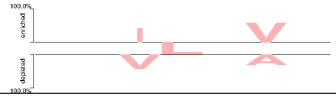		
Spo0B	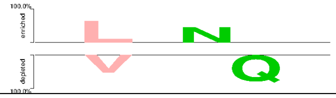		
Spo0A	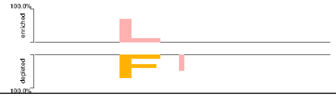	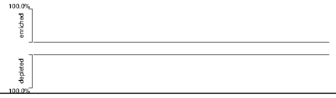	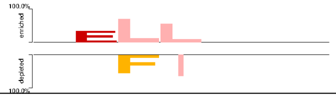

S2Text.Fig. 1: Visual comparison of Spo0 specificity logos, $p < 0.01$

Visual inspection of the logos reveals that phosphorelay specificity residues in the Bacillar and Clostridial phosphorelays are very similar (S2Text.Fig 1, first column). At least half of the sites in each logo comparison have no significant differences in amino acid frequencies. At the majority of sites that do have significant differences, the amino acids with different frequencies have similar biochemical properties; i.e., a hydrophobic amino acid has been replaced with a different hydrophobic amino acid. The greatest difference is observed in the Spo0B specificity residues: asparagine is enriched in Site 3 in the Bacilli and glutamine at site 4 in the Clostridia. The quantitative scores are consistent with these trends (S2Text.Table 1, first column); Spo0B has the greatest per-site difference.

Comparison of the specificity logos of orphan kinases and Spo0A from phosphorelays and direct phosphorylation architectures shows that the similarity across architectures is greater for Spo0A proteins than for orphan kinases (S2Text.Table 1 and S2Text.Fig 1, second and third columns). Spo0A specificity residues are more similar within the same taxonomic class (second column, $S_{CD} = 0.0$), than within the same pathway type (first column, $S_{BC} = 0.29$). The opposite is true for candidate sporulation kinases ($S_{CD} = 0.49$ versus $S_{BC} = 0.18$). The specificity residues of candidate phosphorelay kinases from both the Clostridia and the Bacilli differ markedly from those of kinases predicted to phosphorylate Spo0A directly. The per-site differences in kinase specificity residues across architectures exceed the per-site difference within phosphorelays in Bacilli and Clostridia by more than a factor of two. These results are consistent with architectural remodeling being driven primarily by changes in kinase, rather than Spo0A, specificity.

For comparison, we also generated specificity logos with no p-value filter (i.e., $\alpha = 1$), shown quantitatively and visually in in S2Text.Table 2 and S2Text.Fig 2, respectively. Analysis without filtering shows the raw differences in the distribution of amino acid frequencies observed at each position. The general trends observed in the filtered comparison are also observed without filtering.

S2Text.Table. 2: Quantitative Comparison of Spo0 Specificity Logos, no p-value filtering.

	Clostridial phosphorelay	Clostridial phosphorelay	Bacillar phosphorelay
Spo0 component	Bacillar phosphorelay	Direct Phosphorylation Architecture	
Orphan kinases	0.45	0.73	0.82
Spo0F	0.46		
Spo0B	0.87		
Spo0A	0.69	0.38	0.78

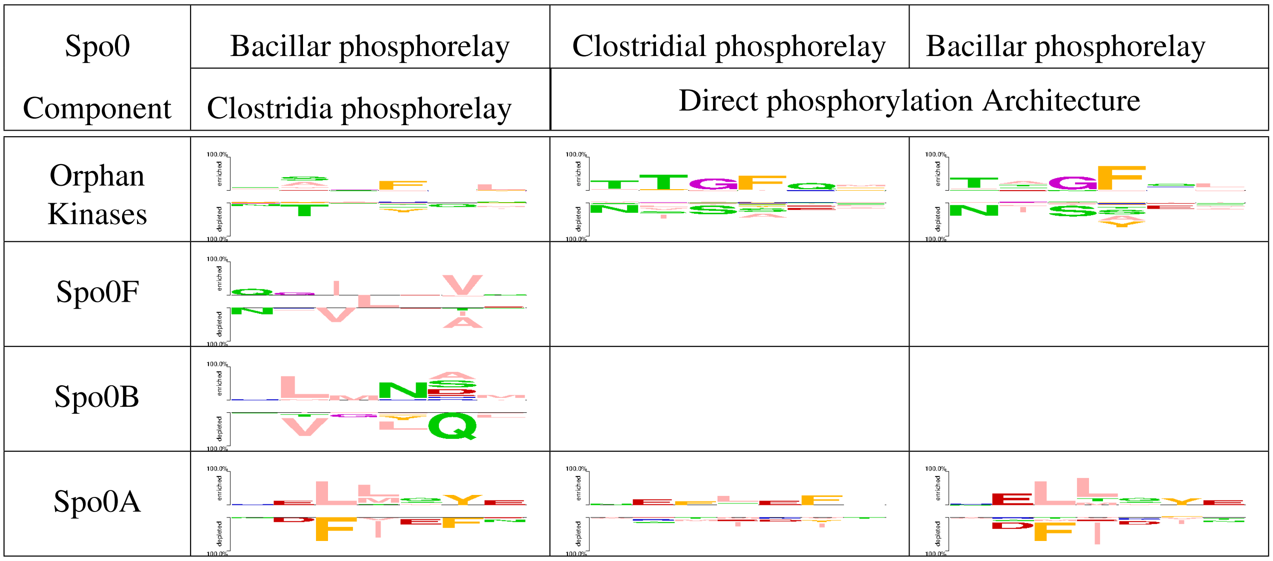

S2Text.Fig. 2: Visual comparison of Spo0 specificity logos, no p-value filtering.

Bibliography

V. Vacic, L. M. Iakoucheva, and P. Radivojac. Two Sample Logo: a graphical representation of the differences between two sets of sequence alignments. *Bioinformatics*, 22(12):1536–7, Jun 15 2006.
