# Supplementary material for: Flexibility and constraint: Evolutionary remodeling of the sporulation initiation pathway in Firmicutes
Source: PLoS Genet. 2018 Sep 13;14(9):e1007470. doi: 10.1371/journal.pgen.1007470 (PMC6136694; doi:10.1371/journal.pgen.1007470)
Supplement: S3 Text — (PDF) [file pgen.1007470.s019.pdf]

S3 Text Comparison of orphan kinase catalytic domain content

The differences between the orphan kinases associated with the two pathway architectures extend beyond specificity residues: the histidine kinase catalytic domains found in the two sets also differ. Histidine kinase catalytic domains have been categorized into 23 subfamilies based on HMM models of multiple sequence alignments [Alexander and Zhulin, 2007]. While many of these catalytic domain subtypes are present in kinases of the Firmicutes, only two, HK_CA:2 and HK_CA:3, are commonly observed in orphan kinases. In genomes with a predicted direct phosphorylation Spo0 pathway, 51 out of 53 orphan kinases (96%) possess a HK_CA:2 type catalytic domain. In genomes with a predicted Spo0 phosphorelay, 130 out of 155 orphan kinases (86%) possess a HK_CA:3 type catalytic domain. The same dichotomy is observed for experimentally verified kinases: direct phosphorylation architecture Spo0 kinases in *R. thermocellum*, *C. difficile*, and, with one exception, *C. acetobutylicum* encode an HK_CA:2 type catalytic domain [Mearls and Lynd, 2014; Steiner et al., 2011; Underwood et al., 2009]. The phosphorelay sporulation kinases in *B. subtilis*, *D. acetoxidans*, and other experimentally verified Spo0 phosphorelays harbor a HK_CA:3 type catalytic domain (see Table S1 for a complete list). The one exception in the experimentally verified set is Ca_C3319 in *C. acetobutylicum*, which encodes an HK_CA:3 domain.

Bibliography

R. P. Alexander and I. B. Zhulin. Evolutionary genomics reveals conserved structural determinants of signaling and adaptation in microbial chemoreceptors. *Proc Natl Acad Sci U S A*, 104(8): 2885–90, Feb 20 2007.

E. B. Mearls and L. R. Lynd. The identification of four histidine kinases that influence sporulation in *Clostridium thermocellum*. *Anaerobe*, 28:109–19, Aug 2014.

E. Steiner, A. Dago, D. Young, J. Heap, N. Minton, J. Hoch, and M. Young. Multiple orphan histidine kinases interact directly with Spo0A to control the initiation of endospore formation in *Clostridium acetobutylicum*. *Mol Microbiol*, 80:641–654, May 2011.

S. Underwood, S. Guan, V. Vijayasubhash, S. Baines, L. Graham, R. Lewis, M. Wilcox, and K. Stephenson. Characterization of the sporulation initiation pathway of *Clostridium difficile* and its role in toxin production. *J Bacteriol*, 191:7296–7305, Dec 2009.
