# Supplementary material for: Flexibility and constraint: Evolutionary remodeling of the sporulation initiation pathway in Firmicutes
Source: PLoS Genet. 2018 Sep 13;14(9):e1007470. doi: 10.1371/journal.pgen.1007470 (PMC6136694; doi:10.1371/journal.pgen.1007470)
Supplement: S4 Text — (PDF) [file pgen.1007470.s020.pdf]

Construction of expression vectors for N-terminal fusion proteins

Expression plasmids for 11 Spo0 proteins from 3 species were constructed using Gateway Cloning Technology (Invitrogen*, https://tools.thermofisher.com/content/sfs/manuals/gatewayman.pdf*). These constructs are summarized in Table 2, reproduced here for the convenience of the reader.

Oligonucleotides encoding *D. acetoxidans* and *C. acetobutylicum* Spo0 proteins were designed and synthesized with codon-usage appropriate for expression in *E. coli* (see Methods). The *B. subtilis* Spo0 proteins were encoded using native nucleic acid sequences derived from the *Bacillus subtilis* subsp. *subtilis* str. 168 genome. These 11 nucleotide sequences are provided in supplementary file S1_Sequence.fasta.

Table 2. Protein Expression Constructs

Protein Name	Locus id	Residues in Spo0 protein		Tag	Fusion Protein Size	
		Start	Stop		residues	kDa
Dt1918	Dtox_1918[1]	301	535	His_6-MBP-TEV	652	72.0
Dt0F	Dtox_0F	1	133	TRX-His_6-TEV	283	31.5
Dt0B	Dtox_0B	1	195	His_6-MBP-TEV	613	68.0
Dt0A	Dtox_0A[2]	1	134	His_6-thrombin	171	18.5
BsKinA	Bsub_KinA	1	606	His_6-MBP-TEV	1024	115.0
Bs0F	Bsub_0F	1	124	TRX-His_6-TEV	274	31.0
Bs0B	Bsub_0B	1	192	TRX-His_6-TEV	342	38.5
Bs0A	Bsub_0A	1	267	TRX-His_6-TEV	417	46.7
Ca0903	CA_C0903[3]	244	683	His_6-MBP-TEV	857	95.0
Ca3319	CA_C3319	1	445	His_6-MBP-TEV	863	95.5
Ca0A	CA_C0A	1	281	His_6-thrombin	318	36.0

To increase protein solubility and stability, the constructs for Dtox_1918, Dtox_0A, and CA_C0903 encode truncated sequences that contain their respective interaction domains. [1]: The N-terminal sensing domains were removed. [2]: REC domain only. [3]: N-terminal trans-membrane region was removed. His_6: hexahistidine sequence; MBP: maltose-binding proteins; -TEV: Tobacco Etch Virus nuclear-inclusion-a endopeptidase cleavage site; TRX: thioredoxin domain; thrombin: thrombin protease cleavage site.

Gateway is a two-step system for high-throughput construction of expression plasmids based on site-specific recombination mediated by a bacteriophage λ integrase [Walhout *et al.,* 2000]. A protein coding sequence is first cloned into an entry vector and then recombined into a destination vector. Given a library of destination vectors that encode a variety of affinity tags, Gateway provides a modular system for rapid construction of expression plasmids with different tag-ORF combinations.

In this study, the Gateway recombinational cloning system was used to fuse an affinity tag to each Spo0 sequence as described by Laub *et al.* [2007]. First, entry clones for all 11 Spo0 sequences were constructed using Gateway's pENTR/D-TOPO entry vector, which allows for topoisomerase-mediated directional cloning (*https://assets.thermofisher.com/TFS-Assets/LSG/manuals/pentr_dtopo_man.pdf*). Next, each Spo0 sequence was transferred to one of three destination vectors encoding an N-terminal affinity tag. Each of these N-terminal sequences encodes a hexahistidine motif and a protease cleavage site. Two of these encode an additional protein domain, either thioredoxin (TRX) or maltose-binding protein (MBP), to enhance the stability of protein folding. The construction of these destination vectors is described in Skerker *et. al.* [2005].

This procedure resulted in 11 expression plasmids, each encoding a Spo0 protein fused to an N-terminal affinity tag under control of an IPTG-inducible promoter. In addition to the affinity tag, the expression plasmids contain sequence elements that are required for topoisomerase-mediated directional cloning, used to construct the entry vectors, and λ integrase-mediated recombinational cloning, used to move the ORF from entry to destination vector. The N-terminal sequences encoded in the expression plasmids are described in detail below. Briefly, each Gateway construct encodes 8 residues that correspond to the core λ integrase recombination site (see attB1 in Figure 1D in Hartley *et al*, [2000]). This sequence is flanked by the sequence upstream (5') of the recombination site in the destination vector and the sequence downstream (3') of the recombination site in the entry vector. The upstream sequences contributed by the destination vectors vary in length and composition, depending on the specific affinity tag used. The downstream sequence contributed by pENTR/D-TOPO entry vector includes a Not1 restriction site, the topoisomerase binding site, and the 4-base pair overhang required for directional cloning. When translated, this introduces a 7-residue linker sequence between the affinity tag and the sequence of the Spo0 protein.

The full length amino acid sequences of the fusion proteins encoded by the resulting expression clones are provided in supplementary file S2_Sequence.fasta.

Amino acid sequences of N-terminal tags

TRX-His$_6$-TEV

MSDKIIHLTDDSFDTDVLKADGAILVDFWAEWCGPCKMIAPILDEIADEYQGKLTVAKLNIDQNPGTAPKYGIRGIP
TLLLFKNGEVAATKVGALSKGQLKEFLDANLAGSGSGHM*HHHHHH*SSG**ENLYFQG**AMI***TSLYKKAG***SAAAPFT

This fusion sequence encodes a thioredoxin domain (underlined), a hexahistidine sequence (*italics*), and a Tobacco Etch Virus nuclear-inclusion-a endopeptidase (TEV) cleavage site (**bold**). These are separated by short linker sequences. The core attB1 site is shown in ***bold italics*** and the 7 residues contributed by pENTR/D-TOPO in red. In total, this N-terminal tag is 150 residues long and increases the weight of the protein by 16 kDa.

His$_6$-MBP-TEV

MK*HHHHHH*PMKIEEGKLVIWINGDKGYNGLAEVGKKFEKDTGIKVTVEHPDKLEEKFPQVAATGDGPDIIFWAH
DRFGGYAQSGLLAEITPDKAFQDKLYPFTWDAVRYNGKLIAYPIAVEALSLIYNKDLLPNPPKTWEEIPALDKELKAK
GKSALMFNLQEPYFTWPLIAADGGYAFKYENGKYDIKDVGVDNAGAKAGLTFLVDLIKNKHMNADTDYSIAEAAF
NKGETAMTINGPWAWSNIDTSKVNYGVTVLPTFKGQPSKPFVGVLSAGINAASPNKELAKEFLENYLLTDEGLEAV
NKDKPLGAVALKSYEEELAKDPRIAATMENAQKGEIMPNIPQMSAFWYAVRTAVINAASGRQTVDEALKDAQTN
SSSNNNNNNNNNNPMS**ENLYFQG**AMI***TSLYKKAG***SAAAPFT

This fusion sequence encodes a hexahistidine sequence (*italics*), the maltose-binding protein (underlined), and a TEV cleavage site (**bold**), separated by short linker peptides. The core attB1 site is shown in ***bold italics*** and the 7-residue sequence contributed by pENTR/D-TOPO in red. In total, this N-terminal tag is 418 residues in length and increases the weight of the protein by 45 kDa.

His$_6$-thrombin

MGSS*HHHHHH*SSG**LVPRGS**HNQ***TSLYKKAG***SAAAPFT

This fusion sequence encodes a hexahistidine sequence (*italics*) and a thrombin protease cleavage site (**bold**). The core attB1 site is shown in ***bold italics*** and the 7-residue sequence contributed by pENTR/D-TOPO in red. In total, this N-terminal tag is 37 residues long and increases the weight of the protein by 3 kDa.

References

Walhout AJM, Temple GF, Brasch MA, Hartley JL, Lorson MA, van den Heuvel S, Vidal M . GATEWAY recombinational cloning: application to the cloning of large numbers of open reading frames or ORFeomes. Methods Enzymol. 2000;328:575-92.

Laub MT, Biondi EG, Skerker JM. Phosphotransfer profiling: systematic mapping of two-component signal transduction pathways and phosphorelays. Methods Enzymol. 2007;423:531-48.

Skerker JM, Prasol MS, Perchuk BS, Biondi EG, Laub MT. Two-component signal transduction pathways regulating growth and cell cycle progression in a bacterium: a system-level analysis. PLoS Biol. 2005 Oct;3(10):e334. Epub 2005 Sep 27.

Hartley JL, Temple GF, Brasch MA. DNA cloning using *in vitro* site-specific recombination. Genome Res. 2000 Nov;10(11):1788-95.

Landy A. The λ integrase site-specific recombination pathway. Microbiol Spectr. 2015 Apr;3(2):MDNA3-0051-2014. doi: 10.1128/microbiolspec.MDNA3-0051-2014.
